# Supplementary material for: Hybrid simulation modelling of networks of heterogeneous care homes and the inter-facility spread of Covid-19 by sharing staff
Source: PLoS Comput Biol. 2022 Jan 12;18(1):e1009780. doi: 10.1371/journal.pcbi.1009780 (PMC8789158; doi:10.1371/journal.pcbi.1009780)
Supplement: S1 Appendix — Table A. The state variables of care homes agents and bank/agency staff agents. Fig A. The process overview and scheduling of the model at each time step. (White box: sub-model; sky-blue box: starting at time step 1; green box: starting at time step 91) Note: FoI–Force of infection. Fig B. The progression of COVID-19 infections. Susceptible people may acquire the infection when exposed to infectious sources. They are infected but not yet infectious (exposed state). Once exposed people become infectious, they can either remain asymptomatic for the entire infectious period or develop symptoms after a pre-symptomatic period. Symptoms could be mild or severe and require hospitalizations. Infectious people will eventually recover or die. Table B. Resident population size and staffing level in care homes within a network. Table C. Initial values of entities’ state variables and parameters. Table D. Parameters used in the model. Fig C. The structure of Intra-facility module embedded in each care home agent. This sub-model developed using stochastic system dynamics represents the transmission dynamics of COVID-19 within a care home. Dash red, blue, and green lines represent transmissions caused by infectious permanent staff, residents, and temporary bank/agency staff respectively. Table E. Summary of equations for stocks and flows in the Intra-facility sub-model. (DOCX) [file pcbi.1009780.s001.docx]

**S1. Appendix. The ODD Protocol**

### Purpose and Patterns

The purpose of the model is to investigate the impacts of staff working across different care homes as well as interventions to mitigate these impacts. The examined interventions include reducing or halting the use of bank/agency staff, weekly PCR testing of bank/agency staff, and creating bubbles of care homes. Care home bubbles restrict bank/agency staff to work only within a specific group of care homes that are designated as one bubble. We build confidence in our model by its ability to reproduce the following patterns observed in care homes in the UK:

- Pattern i: The higher risk of infection for residents and staff in care homes using bank/agency staff frequently compared with those care homes not using bank/agency staff [1]
- Pattern ii: The higher risk of infection for bank/agency staff compared with permanent staff in care homes using bank/agency staff frequently [1,2]
- Pattern iii: The risk of outbreaks in care homes using bank/agency staff frequently compared with those care homes not using bank/agency staff [3]
- Pattern iv: The risk of outbreak occurrence in care homes specified by their resident population size and staff-to-resident ratio [4,5]

Pattern i, ii, and iii which reflect the impact of agency/bank staff use upon the spread of COVID-19 across care homes within a network, are important to clarify that our model is useful for its purposes. Pattern i, ii, and iii help validate the behaviours of the overall system. Pattern iv addresses the validity of the sub-systems’ behaviour (care homes) when accounting for their interactions via bank/agency staff.

### Entities, State Variables, and Scales

The following entities are included in the model: two types of agents, namely care homes and bank/agency staff agents, respectively representing the care homes and temporary bank/agency staff who work in more than one care home within the network. Each agent entity is characterized by a unique set of state variables which are described in greater detail in Table A. A system dynamic (SD) module embedded in each care home agent represents the intra-facility transmission dynamics of COVID-19.

The model runs at a daily time step as epidemiological data are collected on a daily basis and the unit of time commonly used to describe clinical characteristics of COVID-19 in the literature is a day. The modules exchange information daily to capture transmission dynamics across care homes. As bank/agency staff are rostered daily, it is important to update their infection state and the state of SD modules in affected care homes on this time scale. Simulations are 90-day time steps long as this covers the period for planning response strategies to contain the spread of COVID-19.

**Table A. The state variables of care homes agents and bank/agency staff agents**

| **Variable name** | **Variable type, units and range** | **Meaning and rationale** |
| --- | --- | --- |
| **Care home agent specific state variables** | | |
| ID | Integer, static; no unit; > 0 | The identity of the care home |
| GroupID | Integer, static; no unit; > 0 | The identity of the care home sub-group to which a care home belongs |
| N_R_ | Integer, static; residents; > 0 | The capacity of the care home |
| N_S_ | Integer, static; staff members; > 0 | The number of permanent staff members of the care home |
| N_W_ | Integer, static; staff members; > 0 | The desired number of staff members on duty per day when the home operates at full capacity |
| N_U_ | Integer, dynamic; staff members; ≥ 0 | The daily number of unfilled staff positions |
| N_B_ | Integer, dynamic; staff members; ≥ 0 | The daily number of bank/agency staff members working in the care home |
| I_B_ | Integer, dynamic; staff member; ≥ 0 | The daily number of bank/agency staff member that are infectious working in the care home |
| S_B_ | Integer, dynamic; staff member; ≥ 0 | The daily number of bank/agency staff member that are susceptible working in the care home |
| Intra-Facility Module | System dynamic module including the following stocks:  S_R_, E_R_, I_R,_ Q_R,_ R_R_: susceptible, exposed, infectious, isolated, recovered residents  S_S_, E_S_, I_S,_ Q_S,_ R_S_: susceptible, exposed, infectious, isolated, recovered permanent staff  E_SD_, I_SD_: exposed and infectious permanent staff who have been tested and will be detected by PCR testing. They will self-isolate when testing results return. | The transmission dynamics within the care home. The levels of stocks in the SD module are the number of residents and permanent staff in different states of health and disease. |
| **Bank/agency staff agent specific state variables** | | |
| ID | Integer, static; no unit; > 0 | The identity of a staff member in the bank/agency staff pool among care homes |
| GroupID | Integer, static; no unit; > 0 | The identity of the care home bubble to which a bank/agency staff member belongs. The staff member can only work at the care homes with the same GroupID. |
| WorkID | Integer, dynamic; no unit; ≥ 0  0 = Not at work yet | The identity of the care home where a bank/agency staff member works |
| WorkRecord | Array [i]: integers, dynamic; no unit;  i ∈ [1, 2, …] | The work record of a bank/agency staff member across care homes  i = care homes’ ID  WorkRecord [i] = the number of times that the staff member works in care home i |
| InfectionState | Integer; dynamic; no unit;  0 = susceptible  1 = exposed  Infectious (  2 = asymptomatic  3 = pre-symptomatic  4 = symptomatic)  5 = recovered | The state of infection of a member of bank/agency staff |
| Tested | Boolean, dynamic; no unit; true/false | Indicates whether a bank/agency staff member has a RT-PCR test in the last 7 days |
| Isolation | Boolean, dynamic; no unit; true/false | Indicates whether a bank/agency staff member is self-isolating because of having COVID-19 |

### Process Overview and Scheduling

In the warm-up period of 90 days (time step 1 to 90) without infections, the model only executes the process *Temporary_Staff_Schedule,* excluding the sub-model *Staff_Resident_Contact_Rate,* to establish the frequency distribution for bank/agency staff’s work history (i.e.*WorkRecord*). From day 91, the model executes six actions in the following order at each time step (Fig A).


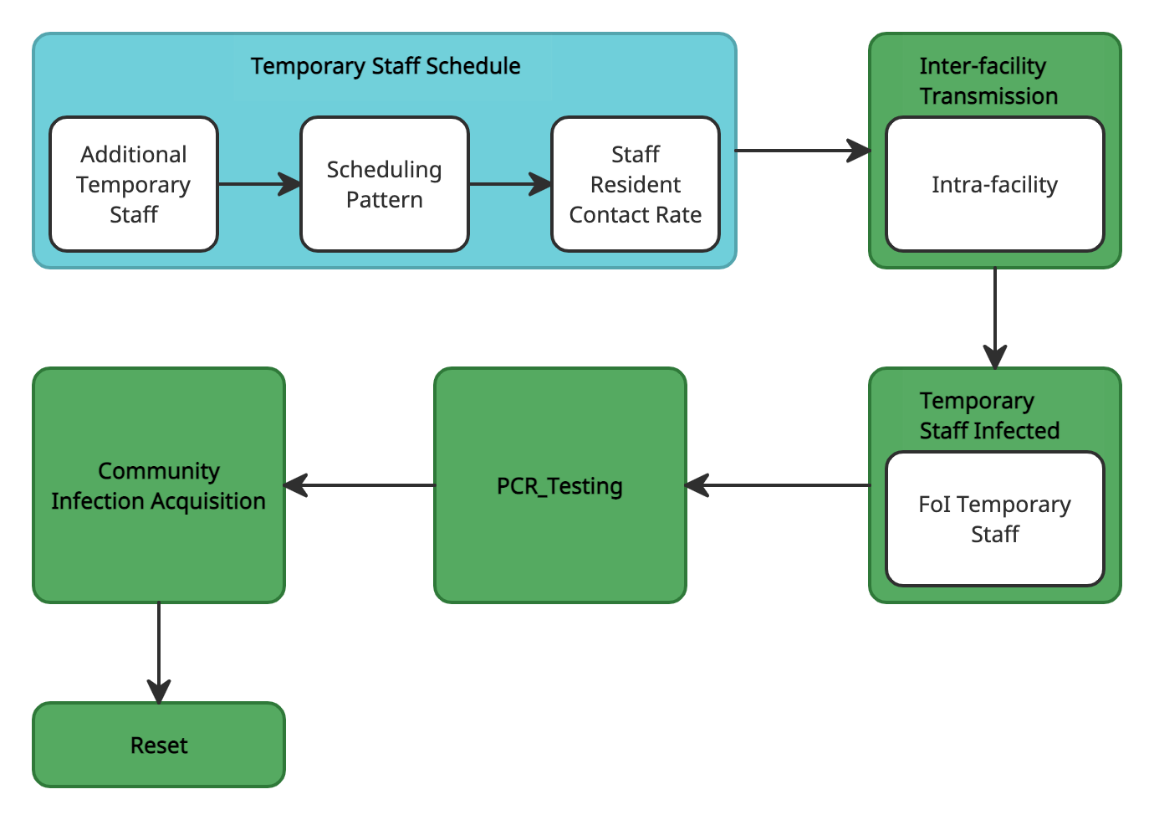


**Fig A. The process overview and scheduling of the model at each time step**

(White box: sub-model; sky-blue box: starting at time step 1; green box: starting at time step 91)

Note: FoI – Force of infection

#### Temporary_Staff_Schedule

This process is executed for every care home agent in random order. Care home *i* seeks to recruit *N_B,i_* bank/agency staff each day calculated as in the sub-model *Temporary_Staff_Requirement.*

The daily required bank/agency staff members are chosen based on one of the patterns described in the sub-model *Scheduling_Pattern.* Their *WorkID* changes to the *ID* of the care home. Among these daily bank/agency staff members, the number of susceptible and infectious ones are respectively used to update the state variables *S_B_* and *I_B_* of the care home agent.

The number of unfilled staff positions determines the state variable *N_U_* of the care home. The daily number of contacts with residents per on-duty staff member (*c_SR_)* are recalculated as described in the sub-model *Staff-Resident_Contact_Rate* due to changes in the daily staff-per-resident ratio.

#### Intra-facility_Transmission

The transmission of COVID-19 occurring within each care home is described in the sub-model *Intra-facility.*

#### Temporary_Staff_Infected

The daily rate at which susceptible bank/agency staff agents at work (*WorkID* $\neq$ 0) acquire the infection is calculated as described in the sub-model *FoI_Temporary_Staff*. The bank/agency staff members who are infected have their state variable *InfectionState* changed from ‘susceptible’ to ‘exposed’.

***Temporary_Staff_Disease_Progression***

Exposed 🡪 Infectious (Pre/Asymptomatic): Exposed staff agents transit to either the pre-symptomatic state at the probability *δ_S_* or the asymptomatic state at the end of the exposure period *τ_e_.*

Within Infectious (Presymptomatic 🡪 Symptomatic): Pre-symptomatic staff agents transit to the symptomatic state when they develop symptoms at the end of their pre-symptomatic period *τ_p_.* Symptomatic staff have to self-isolate at home (*Isolation* = ‘true’) in the next time step until they recover.

Infectious 🡪 Recovered: Infectious staff agents (*InfectionState* = 2,3, or 4) recover at the probability (1 – *d_S_*) at the end of their infectiousness period (*τ_i_*). Their infection state changes to ‘recovered’ and the variable *Isolation* is set to ‘false’. Staff that do not recover are deceased, and they are replaced by new susceptible bank/agency staff agents.

***PCR_Testing***

Bank/agency staff have RT-PCR testing at the interval *τ_pcr_,* starting from the time step 95. Those staff members who are tested at the probability determined by their adherence to testing *ρ_B,pcr_* have their state variable *Tested* set to ‘true’. After the time delay from testing to test result determined by the parameter *τ_trt_*, the infected staff members whose *Tested* is ‘true’ are detected at the probability *θ_pcr_* and have to self-isolate. Their state variable *Tested* is reset to ‘false’.

#### Community_Infection_Acquisition

Bank/agency staff members can also acquire COVID-19 from interactions with other infectious people in the community at the probability *β_C_.*

***Reset***

The variable *WorkID of a*ll bank/agency staff agents is reset to zero, indicating that they leave the workplace at the end of the day. Staff agents who leave the workforce at the turnover rate *µ_S_* will have all of their state variables except *ID* reset as in *Initialization*.

1. **Design Concepts**

#### Basic principles:

The model simulates the spread of COVID-19 within a network of care homes via staff who work at multiple facilities. Staff members who work across several care homes (bank/agency staff) can acquire COVID-19 via contacts with other individuals, including residents and staff in one care home and spread the virus to other care homes. They can also contract the infection from the community and spread it to the care homes where they work.

The risk at which susceptible bank/agency staff contract COVID-19 in a care home depends on the transmission dynamics within that facility which is modelled using SD. In the SD module, residents and permanent staff who only work in that care home are grouped into stocks based on their state of infection (susceptible, exposed, infectious, and recovered). Individuals (either residents or staff members) within a stock are assumed to be homogenous. Infections can be imported into the care home by asymptomatic staff acquiring the infection somewhere else. The guidance on controlling COVID-19 in care homes in the UK requires new residents to have two negative tests prior to admission to a care home and compulsory isolation of 14 days upon admission [6,7]. We, therefore, assume that all newly admitted residents are susceptible for simplification.

Fig B describes the progression of COVID-19 infection after transmission occurs based on the current understanding and evidence of clinical characteristics of COVID-19 [8-10]. It is assumed that pre-/asymptomatic individuals are just as likely to transmit infection as symptomatic individuals and recovered people are immune to re-infection in the short term [11].

**Fig B. The progression of COVID-19 infections**

Susceptible people may acquire the infection when exposed to infectious sources. They are infected but not yet infectious (exposed state). Once exposed people become infectious, they can either remain asymptomatic for the entire infectious period or develop symptoms after a pre-symptomatic period. Symptoms could be mild or severe and require hospitalizations. Infectious people will eventually recover or die.


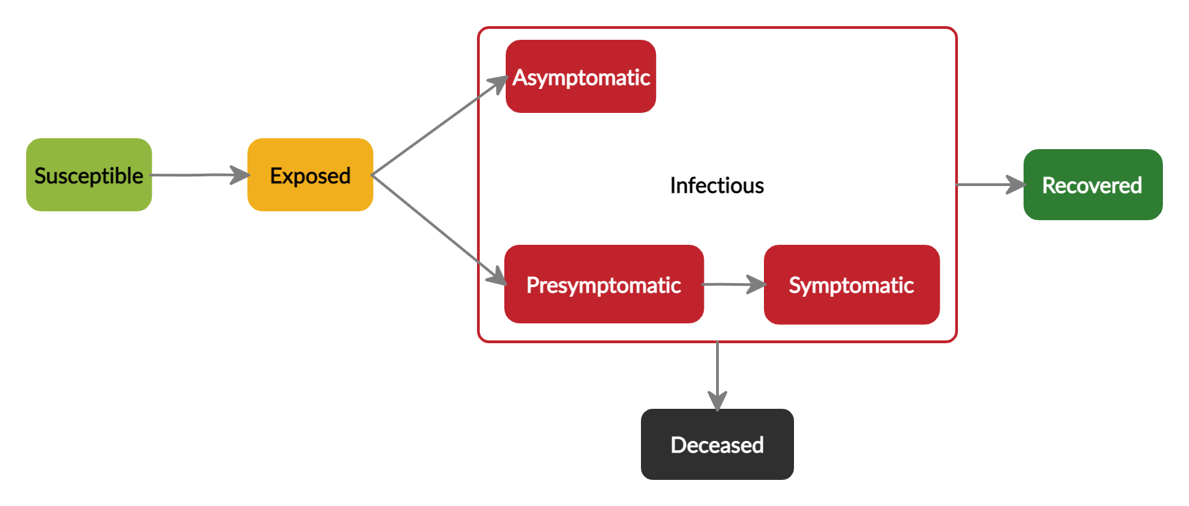


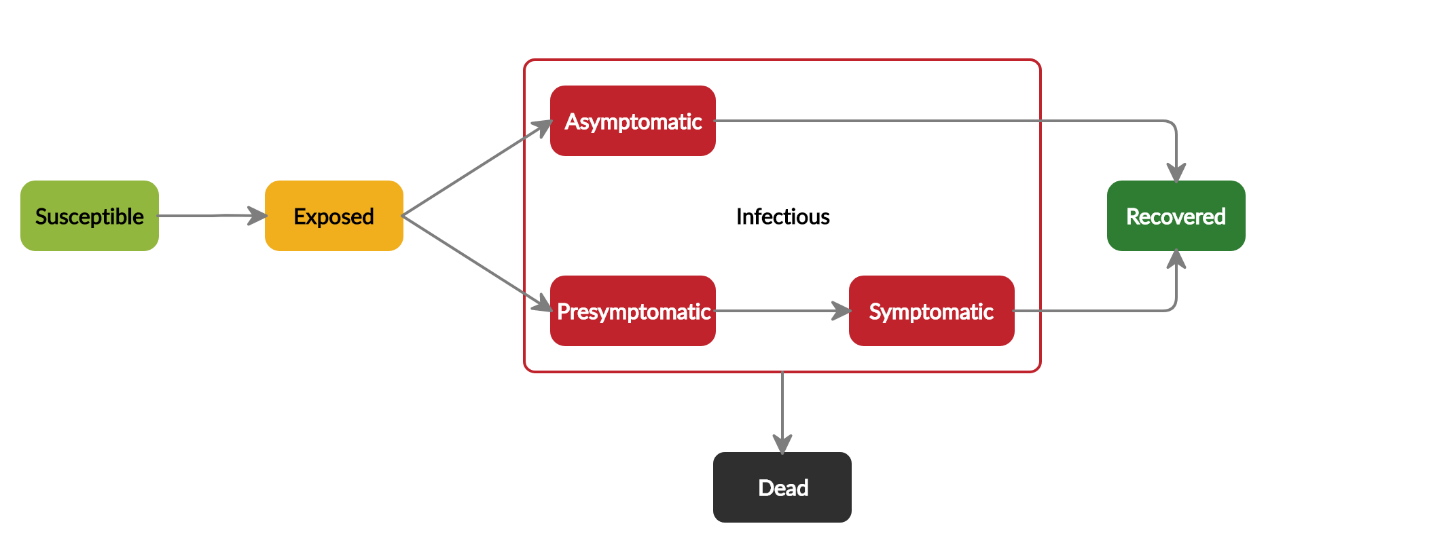
The resident population size, staffing level and operational and managerial features of care homes in the network and how staff are shared among these homes can affect the inter-facility spread of the virus. Such information is obtained through discussions and interviews with stakeholders including Health and Social Care, Council, Public Health and care homes in Lanarkshire, and Scottish Government Data Analysis and Research Group. Infection control measures that target healthcare staff who work at multiple care homes are implemented to contain the inter-facility transmission.

Fig S1-3: The progression of COVID-19 infections

Susceptible people may acquire the infection when exposed to infectious sources. They are infected but not yet infectious (exposed state). Once exposed people become infectious, they can either remain asymptomatic for the entire infectious period or develop symptoms after a pre-symptomatic period. Symptoms could be mild or severe and require hospitalizations. Infectious people will eventually recover or die.

***Emergence:***

The key outcomes of the model are patterns for the occurrence of outbreaks and the scope of affected care homes. These outcomes emerge from the use of bank/agency staff in care homes, infection control interventions targeting this group of staff, infection control measures implemented in care homes, and their resident and staffing characteristics.

#### Adaptation:

Staff that exhibit symptoms or test positive for COVID-19 are required to self-isolate at home. Care homes also isolate their residents who exhibit symptoms. When social distancing is implemented, care homes adapt to the situation by decreasing rates of staff-staff and resident-resident contacts. In intervention scenarios, care homes that experience an outbreak can either increase the use of bank/agency staff to cover the permanent staff members absent due to COVID-19-related reasons. When the intervention of creating bubbles of care homes within which bank/agency staff are restricted to work is implemented in the network, care homes are adaptive to the new situation by using only eligible bank/agency staff members.

#### Objectives:

Objectives are not implemented.

#### Learning:

Learning is not implemented.

#### Prediction:

The staff’s adaptive behaviour is based on implicit predictions that leaving when exhibiting symptoms will disrupt transmission chains in the care home and across care homes. Care homes that continue to operate as normal during an outbreak need to increase their use of bank/agency staff as they expect a shortage of permanent staff absent due to having to self-isolate.

#### Sensing:

Bank/agency staff agents who develop symptoms can sense their own state of health and do not go to work the next day. In intervention scenarios, staff agents can sense which care home(s) they are allowed to work for and, in reverse, care home agents can sense whom they can schedule.

#### Interaction:

Residents can interact with other residents and staff. Staff can interact with other staff members in a care home. The rates of interactions between residents and staff are defined based on the management policy of a care home agent and the implemented infection control interventions such as social distancing. Bank/agency staff agents do not interact with each other outside care homes. Regarding interactions between bank/agency staff and care home agents, bank/agency staff agents are allowed to work in all care homes in the base case and are restricted to work in a bubble of care homes in intervention scenarios.

#### Stochasticity:

Stochasticity is used to describe variability in the parameters that determine the transitions of individuals between different states of infection, including the incubation time and the transmission probability. This represents variations in the risk of acquiring the infection and the progression and outcome of the infection among people, influenced by factors such as their health status, underlying conditions, and immune system. Another stochastic element is contact rates between individuals in a care home that affects the spread of the infection. The movement of bank/agency staff between care homes in the network is also a stochastic process as randomness exists in which care home they come to work on a particular day. This also leads to the stochasticity in the time at which bank/agency staff become infected and introduce the infection into a care home.

#### ***Collectives***:

In intervention scenarios, the model has collectives of care homes and bank/agency staff agents. The collective to which agents belong affects which care homes bank/agency staff can work for – “bubbles”.

#### Observation:

The primary outputs of interest can be observed via plots of the cumulative number of care homes experiencing an outbreak and the cumulative number of infected residents in the entire network over time.

### Initialization

The model is initialized with a network of 12 care homes which consist of a total of 780 residents and 960 permanent staff members (network A). Sizes and staff-to-resident ratios of constituent care homes were initiated once based on the empirical distributions of 84 care homes in Lanarkshire and used for all simulations (Table B). Data in Lanarkshire also reflected the proportions of care homes by size ranges in the UK [1,12]. Network B, C, and D comprise the same number of residents and staff members but have different compositions. Network B consists of homogeneous care homes in terms of size and staff-to-resident ratio. Care homes in network C are homogeneous in size and heterogeneous in staff-to-resident ratio and those in network D have heterogeneous in size and homogeneous in staff-to-resident ratio. Staff under other contract types (bank, agency, temporary, casual, and non-guaranteed hours contracts) constitutes 5 – 20% of total care home staff across various areas in the UK [13-15]. The model is initialized with 107 bank/agency staff agents in base-case simulations (10% of total staff).

**Table B. Resident population size and staffing level in care homes within a network**

| **Network** | **Care Home ID** | **Resident Population Size (N_R_)** | **Total Permanent Staff (N_S_)** |
| --- | --- | --- | --- |
| **Network A** | 1 | 10 | 22 |
|  | 2 | 24 | 31 |
|  | 3 | 32 | 47 |
|  | 4 | 40 | 49 |
|  | 5 | 46 | 73 |
|  | 6 | 50 | 63 |
|  | 7 | 65 | 80 |
|  | 8 | 73 | 90 |
|  | 9 | 80 | 90 |
|  | 10 | 90 | 103 |
|  | 11 | 110 | 110 |
|  | 12 | 160 | 202 |
| **Network B** | 1 – 12 | 65 | 80 |
| **Network C** | 1 | 65 | 50 |
|  | 2 | 65 | 55 |
|  | 3 | 65 | 60 |
|  | 4 | 65 | 65 |
|  | 5 | 65 | 70 |
|  | 6 | 65 | 75 |
|  | 7 | 65 | 80 |
|  | 8 | 65 | 90 |
|  | 9 | 65 | 95 |
|  | 10 | 65 | 100 |
|  | 11 | 65 | 105 |
|  | 12 | 65 | 115 |
| **Network D** | 1 | 10 | 12 |
|  | 2 | 21 | 30 |
|  | 3 | 30 | 39 |
|  | 4 | 40 | 49 |
|  | 5 | 46 | 57 |
|  | 6 | 50 | 63 |
|  | 7 | 65 | 80 |
|  | 8 | 75 | 90 |
|  | 9 | 82 | 98 |
|  | 10 | 92 | 110 |
|  | 11 | 109 | 135 |
|  | 12 | 160 | 197 |

**Table C. Initial values of entities' state variables and parameters**

| **Variable/ Parameter** | **Initial value** | **Source** |
| --- | --- | --- |
| **Care-home-agent-specific state variables** | | |
| ID | 1, 2, …, 12 |  |
| GroupID | 0 |  |
| N_R_ | See Table S1-2 | Provided by Health and Social Care Partnership Lanarkshire |
| N_S_ | See Table S1-2 | Provided by Health and Social Care Partnership Lanarkshire |
| N_W_ | 0.4* N_S_ | Assumption based on discussions with care homes in Lanarkshire |
| N_U_ | 0 |  |
| N_B_ | 0 |  |
| I_B_ | 0 |  |
| S_B_ | 0 |  |
| Intra-facility | S_R_(0) = N_R_; E_R_(0) = I_R_(0) = R_R_(0) = Q_R_(0) = 0  Ss = N_S_; E_S_(0) = I_S_(0) = R_S_(0) = Q_S_(0) = E_SD_(0) = I_SD_(0) = 0 | See the sub-model *Intra-facility* for more details |
| **Shared-staff-agent-specific state variables** | | |
| ID | 1, 2, 3, … |  |
| GroupID | 0 |  |
| WorkID | 0 |  |
| WorkRecord | [0, 0, …, 0] |  |
| InfectionState | 0 (susceptible) |  |
| Tested | False |  |
| Isolation | False |  |

- 1. ***Creating Bubbles of Care homes***

In the scenarios of creating bubbles of care homes, care homes are grouped into *m* bubbles with similar sizes. Care homes can be allocated into bubbles randomly or based on their size or staff-to-resident ratio. The number of bank/agency staff members of bubble *i* ($n_{B,i}$) calculated as $n_{B,i}=\frac{n_{i}}{1-}$

$n_{i}$: Total number of permanent staff in bubble *i*

$n_{B,i}$bank/agency staff agents with *GroupID* = 0 are randomly assigned to group *i* (their *GroupID* change to *i*).

- 1. ***Different Levels of Bank/Agency Staff Use***

When the usage level of bank/agency staff are different from the base-case value (107 bank/agency staff agents – 10% of total staff), the level of permanent staff in each care home and bank/agency staff shared among care homes are adjusted accordingly in the *Initialization* so that the total staff in a network remains constant ($N_{S,total}=1067$).

The number of permanent staff members in care home *i*: $N_{S,i}=\frac{\left( 1- \alpha\right)N_{S, i,base-case}}{\left( 1- \alpha_{base-case} \right)}$

The number of bank/agency staff agents in a network initialized: $\alpha N_{S,total}$

- 1. ***Different Levels of Staff Shortage***

The number of permanent staff members in care home *i* are adjusted in the *Initialization* as $N_{S,i}=\frac{\left( 1- \alpha_{s} \right)N_{S, i,base-case}}{\left( 1- \alpha_{s,base-case} \right)}$ (*α_s_* : the level of staff shortage - % of total staff).

### Input Data

The daily incidence of COVID-19 in the community in the UK during the second wave is used to determine the time series input of the parameter *β_C_* [16]. The model assumes that the undetected cases represent 50% of the total cases in the community [6]. The adjusted incidence is, therefore, calculated by doubling the reported incidence.

1. **Sub-models**

Parameters used in the model are described in Table D.

**Table D. Parameters used in the model**

| Parameter name | Meaning and rationale | Default Value | Sensitivity Analysis | Source |
| --- | --- | --- | --- | --- |
| β_C_ | Incidence rate in the community | Daily incidence in the UK | Triangular distribution (min = 5x10^-5^, max = 0.002, mode = 0.0005) | [16] |
| d_R_ | Infection fatality rate among residents | 35.9% | Triangular distribution (min = 29.1%, max = 43.4%, mode = 35.9%) | [8,17] |
| d_S_ | Infection fatality rate among staff | 0.07% | Triangular distribution (min = 0.003%, max = 0.315%, mode = 0.070%) | [8,17] |
| c_RR_ | The number of contacts that a resident has with other residents per day | 4.1 contacts per resident per day | Triangular distribution (min = 1, max = 5, mode = 4.1) | [18-20] |
| c_SS_ | The number of contacts that a staff has with other staff per day | 9.6 contacts per staff member per day | Triangular distribution (min = 5, max = 15, mode = 9.6) | [18-20] |
| c_RS_ | The daily number of contacts that a resident has with staff per day | 7.9 contacts per resident per day | Triangular distribution (min = 5, max =15, mode = 7.9) | [18-20] |
| c_SR_ | The daily number of contacts that a staff has with residents per day | A function of the daily staff-per-resident ratio | N/A |  |
| µ_S_ | Staff turnover rate | 24% per year | Triangular distribution (min = 14.0%, max = 37.7%, mode = 24.0%) | [14,21] |
| µ_R_ | The rate at which residents leave the care home because of deaths caused by other reasons, moving to another facility, admitted to hospitals, or returning to their own home (rare) | 0.004 deaths or discharges per resident per day | Triangular distribution (min = 0.001, max = 0.005, mode = 0.004) | [12,22] |
| δ_R_ | The probability that an infected resident will develop symptoms | 0.7 | Triangular distribution (min = 0.5, max = 0.9, mode = 0.7) | [8,9] (Based on the age distribution of care home population in the UK) |
| δ_S_ | The probability that an infected staff member will develop symptoms | 0.6 | Triangular distribution (min=0.4, max=0.8, mode=0.6) | [8,9] (For a population like the UK or US) |
| ν | The risk of transmission per susceptible–infectious contact | 0.02 | Triangular distribution (min = 0.001, max = 0.05, mode = 0.02) | [8,23-28] |
| τ_e_ | The time elapsed between first exposure and becoming infectious | 4.6 days | No (This parameter does not significantly affect number of infections as exposed individuals are not infectious. Also, values for this parameter are relatively consistent across studies.) | [29-33] (Lognormal (μ = 1.16, σ = 0.85)) |
| ­τ_p_ | The time elapsed between becoming infectious and onset of symptoms | 2 days | Uniform (1,3) | [4,10,34,35] |
| τ_i_ | The time elapsed between onset of symptoms and recovery (or recovery time for those who remain asymptomatic) | 9.8 days | Lognormal (mean = 9.769, std = 2.44) | [36,37] (Lognormal (μ = 2.249, σ = 0.246)) |
| τ | Isolation period of infected residents and staff | 14 days | N/A | [7] |
| ρ_sd_ | The reduction of resident-resident and staff-staff interactions (i.e. Compliance rate to social distancing) | 0.75 | Triangular distribution (min = 0.2, max = 0.9, mode = 0.75) | Assumed (based on other models’ assumption[8, 38] and discussions with care home staff and managers) |
| ρ_pcr_ | The compliance to routine PCR testing in permanent staff | 0.8 | No (Relative compliance to testing in permanent staff to bank/agency staff is important for the purpose of the model and explored by scenarios) | Scottish Government Data Analysis and Research Group |
| θ_pcr_ | The sensitivity of RT-PCR test | 0.9 | Triangular distribution (min = 0.7, max = 0.98, mode = 0.9) | [39-41] |
| ρ_B,pcr_ | The compliance to routine PCR testing in bank/agency staff | 0.6 | Varied by scenario | Scottish Government Data Analysis and Research Group |
| τ_pcr_ | The interval of routine testing of staff | 7 days | N/A | [7,42] |
| τ_trt_ | The turnaround time of the test | 2 days | Triangular distribution (min = 1, max =4, mode = 2) | Social Care Working Group |
| α | The proportion of bank/agency staff to total staff (i.e. the level of bank/agency staff use) | 10% | Varied by scenario | [13-15] |
| η | The probability that a bank/agency staff member is randomly allocated to a care home | 0.5 | Varied by scenario | Discussion with care home managers and representatives from Public Health Scotland and Health and Social Care Partnership Lanarkshire |

#### Temporary Staff Requirement

#### Care home i seeks to recruit $\boldsymbol{N}_{\boldsymbol{B,i}}$bank/agency staff each day:

####

$$\boldsymbol{N}_{\boldsymbol{B,i}}\boldsymbol{=}\boldsymbol{N}_{\boldsymbol{BN,i}}\boldsymbol{+}\frac{\boldsymbol{Q}_{\boldsymbol{S,i}}\left( \boldsymbol{N}_{\boldsymbol{W,i}}\boldsymbol{-}\boldsymbol{N}_{\boldsymbol{NB,i}} \right)}{\boldsymbol{N}_{\boldsymbol{S,i}}}$$

$$N_{BN,i} \sim Poisson \left( N_{W,i} \right)$$

$N_{NB,i}$describes the number of bank/agency staff required in normal circumstances prior to the COVID-19 pandemic due to ongoing staff shortage and absence of staff for reasons such as holidays, unfilled vacancies, and sickness.

The component $\frac{Q_{S,i} \left( N_{W,i} - N_{NB,i} \right)}{N_{S,i}}$represents the number of bank/agency staff agents required to cover for permanent staff members self-isolating due to COVID-19 ($Q_{S}$).

#### The parameter α is the average percentage usage level of bank/agency staff in all care homes in the network.

#### Scheduling Pattern

Bank/agency staff are chosen from those from those who have not been allocated to any other care home (*WorkID* = 0), are not self-isolating (*Isolation =* false*)*, and belong to the same sub-group of the care home (the same *GroupID*) where they are allocated following two rules one by one until the demand of this care home is fulfilled.

- Rule 1 with probability *η*: A randomly chosen bank/agency staff agent is allocated.
- Rule 2 with probability (1 – *η*): For a care home with the identity number *i* in the network, the bank/agency staff agent with the largest value of *WorkRecord[i]* is allocated.

#### Staff Resident Contact Rate

The daily number of contacts with residents per staff member at work: $c_{SR}= \frac{c_{RS}N_{R}}{N_{W}-N_{U}}$

#### Intra-facility

Fig C describes the structure of the Intra-facility sub-model developed using stochastic SD. This sub-model represents the transmission dynamics within a care home agent. Table C summarizes the equations for stocks of residents and permanent staff in different states of infection and flows between stocks. This module implicitly accounts for the asymptomatic, pre-symptomatic, and symptomatic states via flows ($Flow_{I_{R}Q_{R}}$ and $Flow_{I_{S}Q_{S}}$ ) to keep the structure of the SD module simple, reduce the number of equations, and keep the equations for the transmission flows simple.


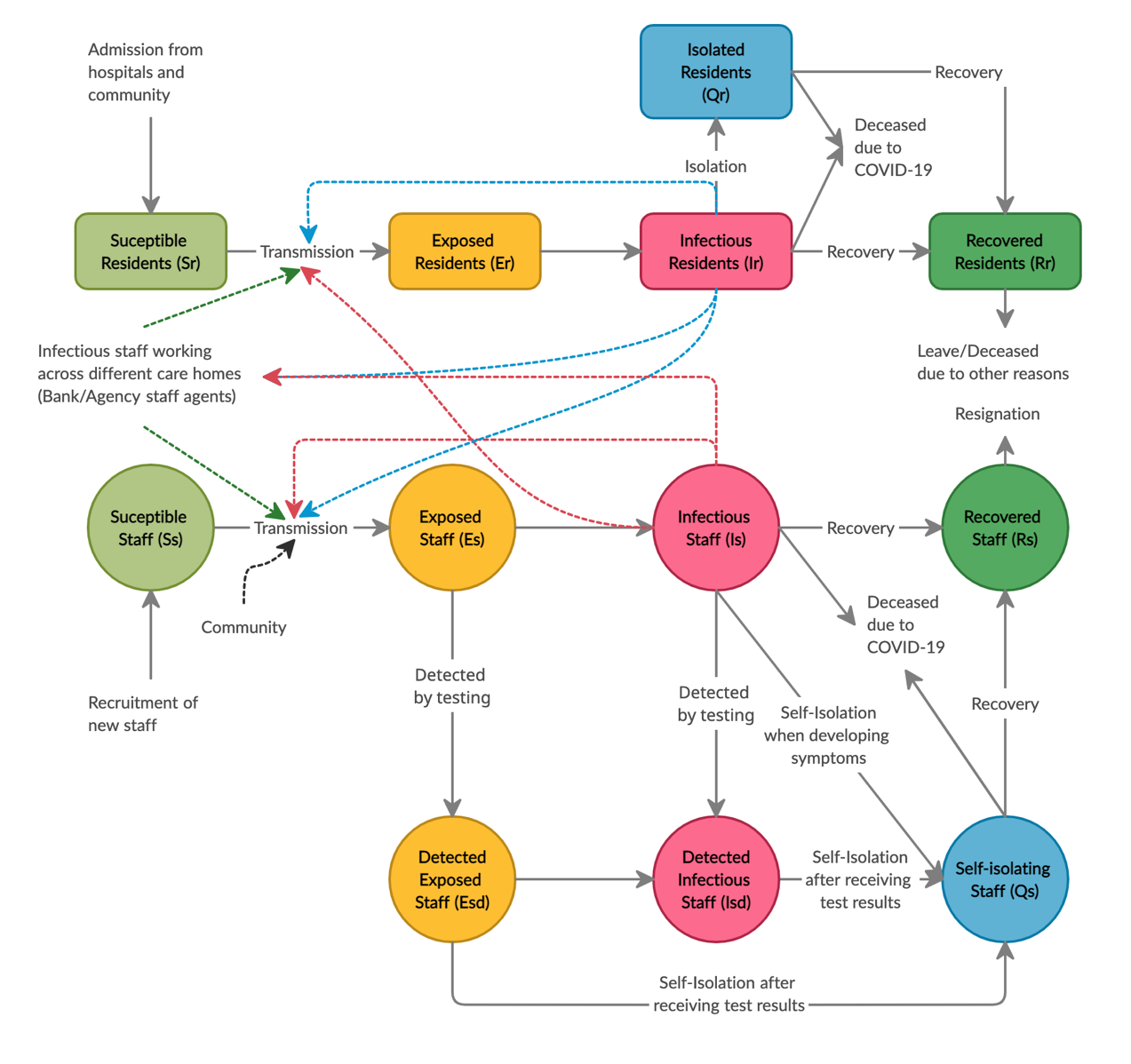


**Fig C. The structure of Intra-facility module embedded in each care home agent**

This sub-model developed using stochastic system dynamics represents the transmission dynamics of COVID-19 within a care home. Dash red, blue, and green lines represent transmissions caused by infectious permanent staff, residents, and temporary bank/agency staff respectively.

**Table E. Summary of equations for stocks and flows in the Intra-facility sub-model**

| **Stock/ Flow** | **Equation** | **Assumption or Comment** |
| --- | --- | --- |
| ***Residents*** | | |
| Susceptible residents | $\frac{d\left( S_{R} \right)}{dt}=Inflow_{S_{R}}-Flow_{S_{R}E_{R}}$ |  |
| Exposed residents | $\frac{d\left( E_{R} \right)}{dt}=Flow_{S_{R}E_{R}}-Flow_{E_{R}I_{R}}$ |  |
| Infectious residents | $\frac{d\left( I_{R} \right)}{dt}=Flow_{E_{R}I_{R}}-Flow_{I_{R}Q_{R}}-Flow_{I_{R}R_{R}}-Outflow_{I_{R}}$ |  |
| Recovered residents | $\frac{d\left( R_{R} \right)}{dt}=Flow_{Q_{R}R_{R}}+Flow_{I_{R}R_{R}}-Outflow_{R_{R}}$ |  |
| Isolated residents | $\frac{d\left( Q_{R} \right)}{dt}=Flow_{I_{R}Q_{R}}-Flow_{Q_{R}R_{R}}-Outflow_{Q_{R}}$ |  |
| Admission of new residents | $Inflow_{S_{R}}=Outlow_{I_{R}}+Outflow_{Q_{R}}+Outflow_{R_{R}}$ | All residents admitted to a care home are susceptible as they receive two compulsory tests and are isolated for 14 days upon admission.[6]  Care homes operate at a full capacity. |
| Transmission to susceptible residents | $Flow_{S_{R}E_{R}}=c_{RR}\left( 1-{}_{sd} \right)\left( 1+F_{1}\xi_{1} \right)S_{R}\frac{I_{R}}{N_{R}}+c_{RS}\left( 1+F_{2}\xi_{2} \right)S_{R}\frac{\left( I_{S}+I_{SD} \right)\left( N_{W}-N_{B} \right)}{N_{S}\left( N_{W}-N_{U} \right)} +c_{RS}S_{R}\frac{I_{B}}{N_{W}-N_{U}}$ | $F_{i}\xi_{i}$: heterogeneous parameter noise, representing parameter fluctuations caused by individual variation (unitless)[43]  $\xi_{i}=\xi_{i}\left( t \right)=\frac{Normal\left( 0,1 \right)}{\sqrt{\delta t}}$  ($\delta$t^-^ = 1/2^7^ days)  $F_{1}=\frac{1}{\sqrt{I_{R}}}$  $F_{2}=\frac{1}{\sqrt{I_{S}+I_{SD}}}$ |
| Residents become infectious | $Flow_{E_{R}I_{R}}=\left( 1+F_{3}\xi_{3} \right)\frac{E_{R}}{{}_{e}}$ | $F_{3}=\frac{1}{\sqrt{E_{R}}}$ |
| Isolation of residents when developing symptoms | $Flow_{I_{R}Q_{R}}={}_{R}\left( 1+F_{1}\xi_{4} \right)\frac{I_{R}}{{}_{p}}$ | We assume perfect effectiveness of resident isolation in the model. |
| Recovery of infected residents | $Flow_{I_{R}R_{R}}=\left( 1-{}_{R} \right)\left( 1-d_{R} \right)\left( 1+F_{1}\xi_{5} \right)\frac{I_{R}}{{}_{i}}$  $Flow_{Q_{R}R_{R}}=DELAY\left( \left( 1-d_{R} \right)Flow_{I_{R}Q_{R}},,0 \right)$ | DELAY(input, delayTime, initialValue): discrete or pipeline. Use of this function means that all delays that it creates take exactly the same length of time, which is delayTime. However, until delayTime is reached, the function will return the initialValue. |
| Death of residents due to COVID-19 | $Outflow_{I_{R}}=\left( 1-{}_{R} \right)d_{R}\left( 1+F_{1}\xi_{6} \right)\frac{I_{R}}{{}_{i}}$  $Outflow_{Q_{R}}=DELAY\left( d_{R}Flow_{I_{R}Q_{R}},,0 \right)$ |  |
| Death/Discharge of residents due to other reasons | $Outflow_{R_{R}}=\mu_{R}R_{R}$ |  |
| ***Staff*** | | |
| Susceptible staff | $\frac{d\left( S_{S} \right)}{dt}=Inflow_{S_{S}}-Flow_{S_{S}E_{S}}$ |  |
| Exposed staff | $\frac{d\left( E_{S} \right)}{dt}=Flow_{S_{S}E_{S}}-Flow_{E_{S}I_{S}}-Flow_{E_{S}E_{SD}}$ |  |
| Exposed staff who are detected by testing | $\frac{d\left( E_{SD} \right)}{dt}=Flow_{E_{S}E_{SD}}-Flow_{E_{SD}I_{SD}}-Flow_{E_{SD}Q_{S}}$ |  |
| Infectious staff | $\frac{d\left( I_{S} \right)}{dt}=Flow_{E_{S}I_{S}}-Flow_{I_{S}Q_{S}}-Flow_{I_{S}R_{S}}-Flow_{I_{S}I_{SD}}-Outflow_{I_{S}}$ |  |
| Infectious staff who are detected by testing | $\frac{d\left( I_{SD} \right)}{dt}=Flow_{I_{S}I_{SD}}+Flow_{E_{SD}I_{SD}}-Flow_{I_{SD}Q_{S}}$ |  |
| Recovered staff | $\frac{d\left( R_{S} \right)}{dt}=Flow_{Q_{S}R_{S}}+Flow_{I_{S}R_{S}}-Outflow_{R_{S}}$ |  |
| Self-Isolating staff | $\frac{d\left( Q_{S} \right)}{dt}=Flow_{I_{S}Q_{S}}+Flow_{E_{SD}Q_{S}}+ Flow_{I_{SD}Q_{S}}-Flow_{Q_{S}R_{S}}-Outflow_{Q_{S}}$ |  |
| Recruitment of new permanent staff to replace staff who have left | $Inflow_{S_{S}}=Outflow_{I_{S}}+Outflow_{Q_{S}}+Outflow_{R_{S}}$ | Permanent staff who leave a care home are replaced by new recruited permanent staff.  All new recruited staff are susceptible. |
| Transmission to susceptible staff | $Flow_{S_{S}E_{S}}=c_{SS}\left( 1-{}_{sd} \right)S_{S}\frac{N_{W}-N_{B}}{N_{S}}\left( \left( 1+F_{2}\xi_{7} \right)\frac{\left( I_{S}+I_{SD} \right)\left( N_{W}-N_{B} \right)}{N_{S}\left( N_{W}-N_{U} \right)}+\frac{I_{B}}{N_{W}-N_{U}} \right)+c_{SR}\left( 1+F_{1}\xi_{8} \right)S_{S}\frac{I_{R}}{N_{R}}\frac{N_{W}-N_{B}}{N_{S}} +{}_{C}\left( 1+F_{4}\xi_{9} \right)S_{S}$ | $F_{4}=\frac{1}{\sqrt{S_{S}}}$ |
| Staff becoming infectious | $Flow_{E_{S}I_{S}}=\left( 1- \right)\left( 1+F_{5}\xi_{10} \right)\frac{E_{S}}{{}_{e}}$  $Flow_{E_{SD}I_{SD}}=\left( 1-k \right)\left( 1+F_{6}\xi_{11} \right)\frac{E_{SD}}{{}_{e}}$ | $F_{5}=\frac{1}{\sqrt{E_{S}}}$  $F_{6}=\frac{1}{\sqrt{E_{SD}}}$  t = 0: {*TestOn = 95;*  *TestReturn = TestON + τ_trt_*}  t = TestON: *θ = θ_pcr_* ρ_pcr_*  t ≠ TestON: *θ = 0*  t = TestReturn: {*k = 1;*  *TestON = TestON + τ_pcr_;*  *TestReturn = TestON + τ_trt_*}  t ≠ TestReturn: *k = 0* |
| Infected staff detected by testing | $Flow_{E_{S}E_{SD}}=E_{S}$  $Flow_{I_{S}I_{SD}}=I_{S}$ |  |
| Self-isolation of staff when developing symptoms or tested positive | $Flow_{I_{S}Q_{S}}=\left( 1- \right)_{S}\left( 1+F_{7}\xi_{12} \right)\frac{I_{S}}{{}_{p}}$  $Flow_{I_{SD}Q_{S}}=\left( 1-k \right)_{S}\frac{I_{SD}}{{}_{p}}+kI_{SD}$  $Flow_{E_{SD}Q_{S}}=kE_{SD}$ | $F_{7}=\frac{1}{\sqrt{I_{S}}}$ |
| Recovery of infected staff | $Flow_{I_{S}R_{S}}=\left( 1- \right)\left( 1-{}_{S} \right)\left( 1-d_{S} \right)\left( 1+F_{7}\xi_{13} \right)\frac{I_{S}}{{}_{i}}$  $Flow_{Q_{S}R_{S}}=DELAY\left( \left( 1-d_{S} \right)\left( {Flow}_{I_{S}Q_{S}}+{Flow}_{E_{SD}Qs} +{Flow}_{I_{SD}Q_{S}} \right),,0 \right)$ |  |
| Death of staff due to COVID-19 | $Outflow_{I_{S}}=\left( 1- \right)\left( 1-{}_{S} \right)d_{S}\left( 1+F_{7}\xi_{14} \right)\frac{I_{S}}{{}_{i}}$  $Outflow_{Q_{S}}=DELAY\left( d_{S}\left( {Flow}_{I_{S}Q_{S}}+{Flow}_{E_{SD}Qs} +{Flow}_{I_{SD}Q_{S}} \right),,0 \right)$ |  |
| Staff turnover | $Outflow_{R_{S}}=\mu_{S}R_{S}$ |  |

#### FoI Temporary Staff

Susceptible bank/agency staff can acquire the infection via contacts with infectious residents and staff members at the care homes where they work at the rate calculated as follow:

$c_{SR}\frac{I_{R}}{N_{R}}+c_{SS}\left( 1-{}_{sd} \right)\frac{I_{W}}{N_{W}-N_{U}}$ (1)

$I_{W}=I_{SW}+I_{B}$ (2)

$I_{SW}=\frac{\left( I_{S}+I_{SD} \right)\left( N_{W}-N_{B} \right)}{N_{S}}$ (3)

In which,

$I_{W}-N_{U}$: The daily number of staff members at work (permanent and bank/agency staff)

$N_{W}-N_{B}$: The daily number of permanent staff members at work

$I_{W}$: The number of infectious staff members at work

$I_{SW}$: The number of infectious permanent staff members at work

Replace (2) & (3) into (1), the rate becomes:

$c_{SR}\frac{I_{R}}{N_{R}}+c_{SS}\left( 1-{}_{sd} \right)\frac{{\frac{\left( I_{S}+I_{SD} \right)\left( N_{W}-N_{B} \right)}{N_{S}}+I}_{B}}{N_{W}-N_{U}}$

**References**

1. Shallcross L, Burke D, Abbott O, Donaldson A, Hallatt G, Hayward A, et al. Factors associated with SARS-CoV-2 infection and outbreaks in long-term care facilities in England: a national cross-sectional survey. The Lancet Healthy Longevity. 2021;2(3):e129-e42.

2. Ladhani SN, Chow JY, Janarthanan R, Fok J, Crawley-Boevey E, Vusirikala A, et al. Increased risk of SARS-CoV-2 infection in staff working across different care homes: enhanced CoVID-19 outbreak investigations in London care Homes. Journal of Infection. 2020.

3. Green R, Tulloch JSP, Tunnah C, Coffey E, Lawrenson K, Fox A, et al. COVID-19 testing in outbreak free care homes: What are the public health benefits? The Journal of hospital infection. 2021.

4. Gatto M, Bertuzzo E, Mari L, Miccoli S, Carraro L, Casagrandi R, et al. Spread and dynamics of the COVID-19 epidemic in Italy: Effects of emergency containment measures. Proceedings of the National Academy of Sciences. 2020;117(19):10484-91.

5. Scottish-Government. Coronavirus (COVID-19): daily data for Scotland. 2020.

6. Scottish Government. National Clinical and Practice Guidance for Adult Care Homes in Scotland during the COVID-19 Pandemic 2020 [cited 2020 2 Dec]. Available from: https://www.gov.scot/binaries/content/documents/govscot/publications/advice-and-guidance/2020/03/coronavirus-covid-19-clinical-and-practice-guidance-for-adult-care-homes/documents/clinical-guidance-for-nursing-home-and-residential-care-residents/clinical-guidance-for-nursing-home-and-residential-care-residents/govscot%3Adocument/National%2BClinical%2BGuidance%2Bfor%2BCare%2BHomes%2BCOVID-19%2BPandemic-%2BMASTER%2BCOPY%2B-%2BFINAL%2B-%2B15%2BMay%2B2020.pdf.

7. Scottish-Government. COVID-19: Information and Guidance for Care Home Settings (Adults and Older People) 2020 [Available from: https://hpspubsrepo.blob.core.windows.net/hps-website/nss/2980/documents/1_covid-19-information-and-guidance-for-care-homes.pdf.

8. Ferguson N, Laydon D, Nedjati Gilani G, Imai N, Ainslie K, Baguelin M, et al. Report 9: Impact of non-pharmaceutical interventions (NPIs) to reduce COVID19 mortality and healthcare demand. 2020.

9. Verity R, Okell LC, Dorigatti I, Winskill P, Whittaker C, Imai N, et al. Estimates of the severity of coronavirus disease 2019: a model-based analysis. The Lancet infectious diseases. 2020.

10. He X, Lau EH, Wu P, Deng X, Wang J, Hao X, et al. Temporal dynamics in viral shedding and transmissibility of COVID-19. Nature medicine. 2020:1-4.

11. Centers for Disease Control and Prevention. COVID-19 Pandemic Planning Scenarios 2020 [Available from: https://www.cdc.gov/coronavirus/2019-ncov/hcp/planning-scenarios.html.

12. ISD. Care Home Census for Adults in Scotland: Figures for 2007-2017 (as at 31 March) 2018 [Available from: https://www.isdscotland.org/Health-Topics/Health-and-Social-Community-Care/Publications/2018-09-11/2018-09-11-CHCensus-Report.pdf.

13. SSSC. Scottish Social Service Sector: Report on 2019 Workforce Data: An Official Statistics Publication for Scotland 2020 [Available from: https://data.sssc.uk.com/data-publications/22-workforce-data-report.

14. Fenton W, Davison S, Polzin G, Price R, McCaffrey R, Fozzard T, et al. The state of the adult social care sector and workforce in England October 2020. Skills for Care: Workforce Intelligence; 2020.

15. Allan S, Vadean F. The impact of workforce composition and characteristics on English care home quality. 2017.

16. GOV.UK. Coronavirus (COVID-19) in the UK 2021 [Available from: https://coronavirus.data.gov.uk/details/cases.

17. Knock E, Whittles L, Lees J, Perez Guzman P, Verity R, Fitzjohn R, et al. Report 41: The 2020 SARS-CoV-2 epidemic in England: key epidemiological drivers and impact of interventions.

18. van den Dool C, Bonten MJM, Hak E, Heijne JCM, Wallinga J. The Effects of Influenza Vaccination of Health Care Workers in Nursing Homes: Insights from a Mathematical Model. PLoS medicine. 2008;5(10):e200.

19. Chamchod F, Ruan S. Modeling the Spread of Methicillin-Resistant Staphylococcus aureus in Nursing Homes for Elderly. PloS one. 2012;7(1):e29757.

20. Simon CP, Percha B, Riolo R, Foxman B. Modeling bacterial colonization and infection routes in health care settings: analytic and numerical approaches. Journal of theoretical biology. 2013;334:187-99.

21. Scottish-Care. Care home workforce data report 2018 [cited 2020 15 August]. Available from: https://scottishcare.org/wp-content/uploads/2019/11/Care-Home-Workforce-Data-2018.pdf.

22. Office for National Statistics. Deaths in the care sector, England and Wales: 2019 2020 [Available from: https://www.ons.gov.uk/peoplepopulationandcommunity/birthsdeathsandmarriages/deaths/bulletins/deathsinthecaresectorenglandandwales/2019.

23. Wang K, Lu Z, Wang X, Li H, Li H, Lin D, et al. Current trends and future prediction of novel coronavirus disease (COVID-19) epidemic in China: a dynamical modeling analysis. Mathematical Biosciences and Engineering. 2020;17(4):3052.

24. Tang B, Bragazzi NL, Li Q, Tang S, Xiao Y, Wu J. An updated estimation of the risk of transmission of the novel coronavirus (2019-nCov). Infectious disease modelling. 2020;5:248-55.

25. Tang S, Tang B, Bragazzi NL, Xia F, Li T, He S, et al. Stochastic discrete epidemic modeling of COVID-19 transmission in the Province of Shaanxi incorporating public health intervention and case importation. medRxiv. 2020.

26. Tang B, Xia F, Bragazzi NL, Wang X, He S, Sun X, et al. Lessons drawn from China and South Korea for managing COVID-19 epidemic: insights from a comparative modeling study. medRxiv. 2020.

27. Zhang AZ, Enns E. Optimal timing and effectiveness of COVID-19 outbreak responses in China: a modelling study. Available at SSRN. 2020.

28. Sun K, Wang W, Gao L, Wang Y, Luo K, Ren L, et al. Transmission heterogeneities, kinetics, and controllability of SARS-CoV-2. Science. 2021;371(6526):eabe2424.

29. Lauer SA, Grantz KH, Bi Q, Jones FK, Zheng Q, Meredith HR, et al. The incubation period of coronavirus disease 2019 (COVID-19) from publicly reported confirmed cases: estimation and application. Annals of internal medicine. 2020.

30. Li Q, Guan X, Wu P, Wang X, Zhou L, Tong Y, et al. Early transmission dynamics in Wuhan, China, of novel coronavirus–infected pneumonia. New England Journal of Medicine. 2020.

31. Qin J, You C, Lin Q, Hu T, Yu S, Zhou X-H. Estimation of incubation period distribution of COVID-19 using disease onset forward time: a novel cross-sectional and forward follow-up study. Available at SSRN 3548756. 2020.

32. McAloon CG, Collins A, Hunt K, Barber A, Byrne A, Butler F, et al. The incubation period of COVID-19: A rapid systematic review and meta-analysis of observational research. medRxiv. 2020.

33. Nishiura H, Linton NM, Akhmetzhanov AR. Serial interval of novel coronavirus (COVID-19) infections. International journal of infectious diseases. 2020.

34. NHS. Rapid Review of the literature: Assessing the infection prevention and control measures for the prevention and management of COVID-19 in health and care settings 2020 [cited 2020 23 May]. Available from: https://www.hps.scot.nhs.uk/web-resources-container/rapid-review-of-the-literature-assessing-the-infection-prevention-and-control-measures-for-the-prevention-and-management-of-covid-19-in-healthcare-settings/.

35. Byrne AW, McEvoy D, Collins A, Hunt K, Casey M, Barber A, et al. Inferred duration of infectious period of SARS-CoV-2: rapid scoping review and analysis of available evidence for asymptomatic and symptomatic COVID-19 cases. medRxiv. 2020.

36. Wölfel R, Corman VM, Guggemos W, Seilmaier M, Zange S, Müller MA, et al. Virological assessment of hospitalized patients with COVID-2019. Nature. 2020;581(7809):465-9.

37. Kerr CC, Stuart RM, Mistry D, Abeysuriya RG, Hart G, Rosenfeld K, et al. Covasim: an agent-based model of COVID-19 dynamics and interventions. medRxiv. 2020.

38. Matrajt L, Leung T. Evaluating the Effectiveness of Social Distancing Interventions to Delay or Flatten the Epidemic Curve of Coronavirus Disease. Emerging infectious diseases. 2020;26(8).

39. Watson J, Whiting PF, Brush JE. Interpreting a covid-19 test result. Bmj. 2020;369.

40. FDA. Accelerated emergency use authorization (EUA) summary COVID-19 RT-PCR test (Laboratory Corporation of America). 2020.

41. Arevalo-Rodriguez I, Buitrago-Garcia D, Simancas-Racines D, Zambrano-Achig P, del Campo R, Ciapponi A, et al. False-negative results of initial RT-PCR assays for COVID-19: a systematic review. medRxiv. 2020.

42. GOV.UK. Guidance Coronavirus (COVID-19) testing for adult social care settings: Department of Health and Social Care; 2021 [Available from: https://www.gov.uk/government/publications/coronavirus-covid-19-testing-for-adult-social-care-settings.

43. Keeling MJ, Rohani P. Modeling Infectious Diseases in Humans and Animals: Princeton University Press; 2008.
