# Supplementary material for: Hybrid simulation modelling of networks of heterogeneous care homes and the inter-facility spread of Covid-19 by sharing staff
Source: PLoS Comput Biol. 2022 Jan 12;18(1):e1009780. doi: 10.1371/journal.pcbi.1009780 (PMC8789158; doi:10.1371/journal.pcbi.1009780)
Supplement: S2 Appendix — Fig A. Comparisons of results generated from parallel system dynamics [SD], stochastic SD, and agent-based [ABM] models. The figure describes the time series of Covid-19 prevalence among residents in care home with capacity of 80 residents (1,000 simulations per scenario). Base-case parameters are used. Interventions implemented in the care home include testing upon admission of residents, no visitation, hand hygiene and using PPE, social distancing, isolation of symptomatic/confirmed residents, and weekly testing of staff. Simulations are seeded with one infected resident. Box-plot: lower hinge: 25% quantile; lower whisker: smallest observation greater than or equal to lower hinge − 1.5×IQR; middle: median; upper hinge: 75% quantile; upper whisker: largest observation less than or equal to upper hinge + 1.5×IQR). Note: IQR–interquartile range. Fig B. Time series of Covid-19 prevalence among residents. (A) and cumulative number of infected residents after 90 days (B) with different values of intra-facility transmission risk. The figure describes the model outcomes for network B in three scenarios: The intra-facility per-contact transmission risk is i/ “Const”: homogeneous across care homes (0.02); ii/ heterogeneous across care homes and drawn from Beta distribution (5, 266); iii/ heterogeneous and drawn from Beta distribution (2, 117). No intervention in bank/agency staff is implemented. Bank/agency staff comprises 10% of total staff. Other parameters have the base-case values. Boxplot: middle–median; lower hinge– 25% quantile; upper hinge– 75% quantile; lower whisker = smallest observation greater than or equal to lower hinge—1.5 * IQR; upper whisker = largest observation less than or equal to upper hinge + 1.5 * IQR. Table A. Relevant studies for black-box validation identified from a systematic search (DOCX) [file pcbi.1009780.s002.docx]

# S2. Appendix. Methods

## Verification

In code verification, we conducted unit testing – all processes and functions were tested individually before incorporating into the main simulation code. For example, we tested creating bubbles of care homes in a simple ABM in which care home agents only had one state variable that defined the identity number of the bubble to which the care homes belonged. This test confirmed the number of care homes per bubble was as intended in the conceptual model before implementing the code in the full simulation. We also performed tracing of randomly chosen agents of each type via the simulation output and used the built-in debugger, bottom-up testing, stress testing, and regression testing for verification.


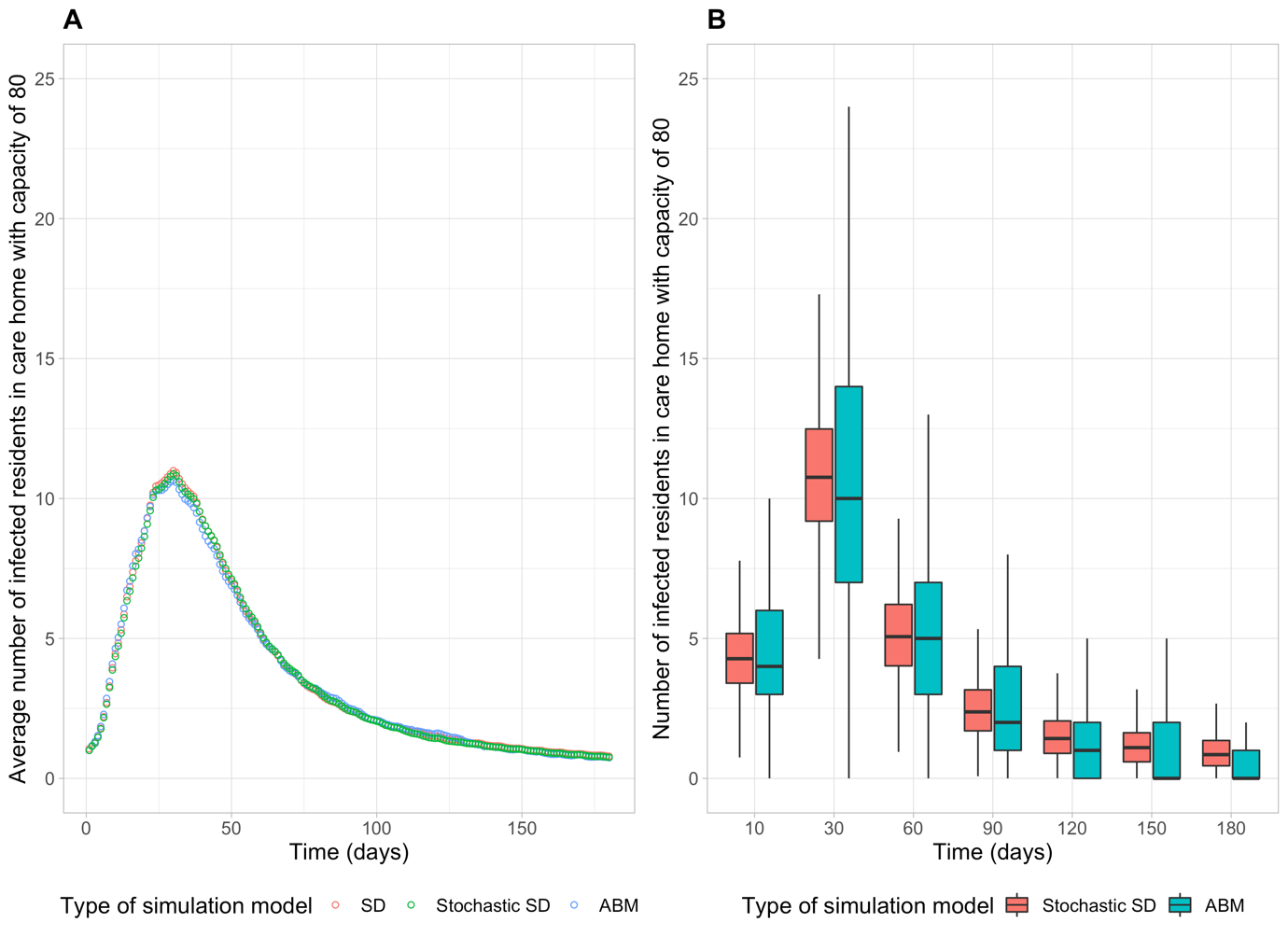


**Fig A. Comparisons of results generated from parallel system dynamics [SD], stochastic SD, and agent-based [ABM] models**

The figure describes the time series of Covid-19 prevalence among residents in care home with capacity of 80 residents (1,000 simulations per scenario). Base-case parameters are used. Interventions implemented in the care home include testing upon admission of residents, no visitation, hand hygiene and using PPE, social distancing, isolation of symptomatic/confirmed residents, and weekly testing of staff. Simulations are seeded with one infected resident.

Box-plot: lower hinge: 25% quantile; lower whisker: smallest observation greater than or equal to lower hinge − 1.5×IQR; middle: median; upper hinge: 75% quantile; upper whisker: largest observation less than or equal to upper hinge + 1.5×IQR). Note: IQR – interquartile range.

## White-box validation


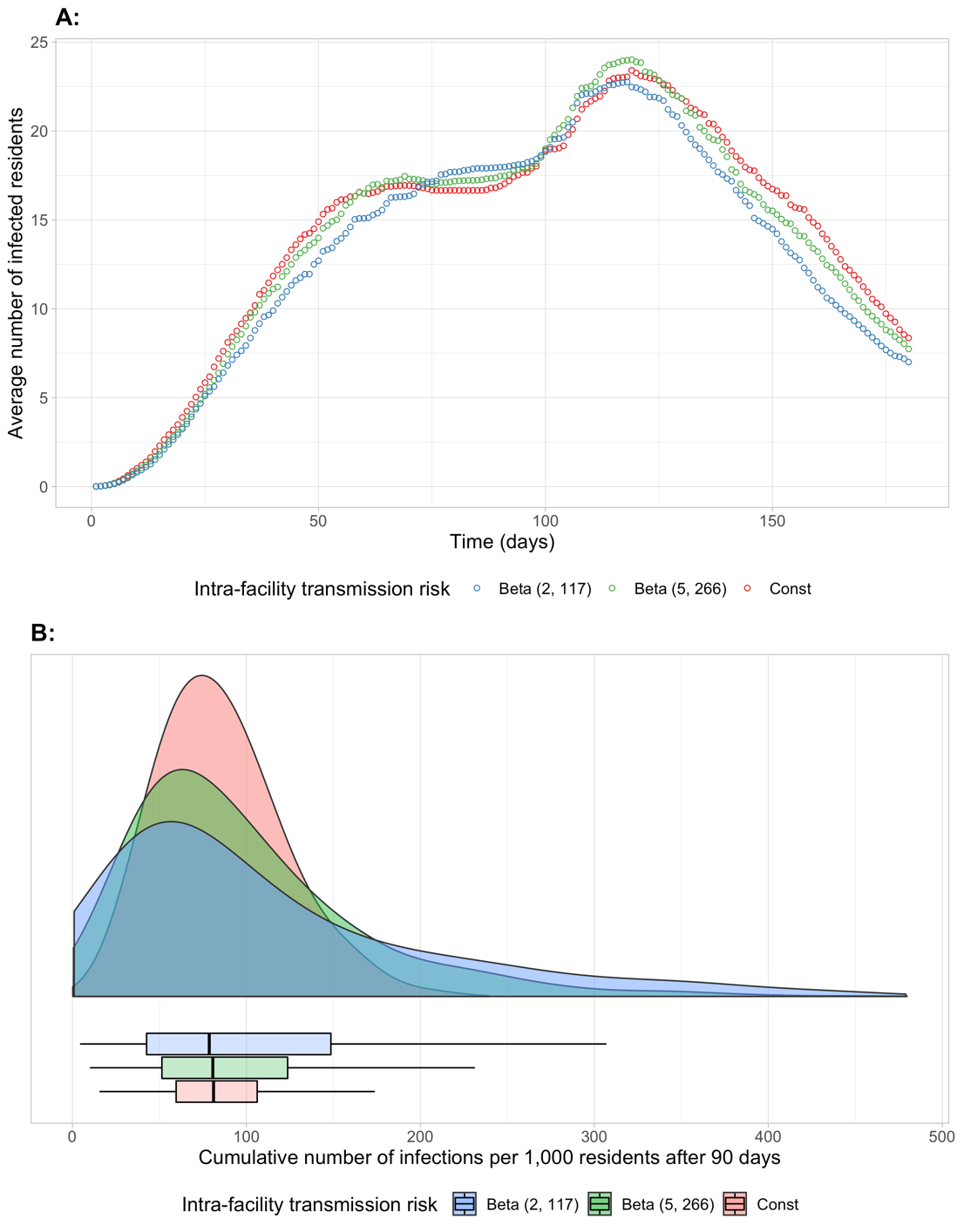


**Fig B. Time series of Covid-19 prevalence among residents**

(A) and cumulative number of infected residents after 90 days (B) with different values of intra-facility transmission risk. The figure describes the model outcomes for network B in three scenarios: The intra-facility per-contact transmission risk is

i/ “Const”: homogeneous across care homes (0.02);

ii/ heterogeneous across care homes and drawn from Beta distribution (5, 266);

iii/ heterogeneous and drawn from Beta distribution (2, 117).

No intervention in bank/agency staff is implemented. Bank/agency staff comprises 10% of total staff. Other parameters have the base-case values. Boxplot: middle – median; lower hinge – 25% quantile; upper hinge – 75% quantile; lower whisker = smallest observation greater than or equal to lower hinge - 1.5 * IQR; upper whisker = largest observation less than or equal to upper hinge + 1.5 * IQR.

## Literature review

We searched PubMed, the WHO Covid-19 database, and medRxiv on the 25^th^ June 2021, with the search terms (“COVID-19” OR “SARS-CoV-2” OR “coronavirus”) AND (“care home*” OR “LTCF*” OR “long term care” OR “nursing home*”) AND (“staff*” OR “healthcare worker*” OR “outbreak*”). These searches returned 306 studies, of which five examined the impact of staff working across multiple care homes on the inter-facility transmission or the impact of care home characteristics including resident population size and staff-to-resident ratio on the risk of outbreak occurrence. In addition to these searches, we identified relevant information from daily COVID-19 data for Scottish and English care homes. Table A below listed the studies to which we compared our modelling results.

**Table A. Relevant studies for black-box validation identified from a systematic search**

| Baister M, McTaggart E, McMenemy P, Megiddo I, Kleczkowski A. COVID-19 in Scottish care homes: A metapopulation model of spread among residents and staff. *medRxiv.* 2021:2021.2008.2024.21262524. (Added on the 30^th^ August) |
| --- |
| Burton JK, Bayne G, Evans C, et al. Evolution and impact of COVID-19 outbreaks in care homes: population analysis in 189 care homes in one geographic region. *medRxiv.* 2020:2020.2007.2009.20149583. |
| Green R, Tulloch JSP, Tunnah C, et al. COVID-19 testing in outbreak free care homes: What are the public health benefits? *The Journal of hospital infection.* 2021. |
| Ladhani SN, Chow JY, Janarthanan R, et al. Increased risk of SARS-CoV-2 infection in staff working across different care homes: enhanced CoVID-19 outbreak investigations in London care Homes. *Journal of Infection.* 2020. |
| Scottish Government. Coronavirus (COVID-19): daily data for Scotland. <https://www.gov.scot/publications/coronavirus-covid-19-daily-data-for-scotland/> Published 2020. Accessed 25 June, 2021. |
| Shallcross L, Burke D, Abbott O, et al. Factors associated with SARS-CoV-2 infection and outbreaks in long-term care facilities in England: a national cross-sectional survey. *The Lancet Healthy Longevity.* 2021;2(3):e129-e142. |
