# Supplementary material for: Hybrid simulation modelling of networks of heterogeneous care homes and the inter-facility spread of Covid-19 by sharing staff
Source: PLoS Comput Biol. 2022 Jan 12;18(1):e1009780. doi: 10.1371/journal.pcbi.1009780 (PMC8789158; doi:10.1371/journal.pcbi.1009780)
Supplement: S3 Appendix — Table A. Risk of infection in residents and staff in various usage levels of bank/agency staff. Fig A. Relative risk [RR] of infection for bank/agency staff with different compliance rates to weekly PCR testing to permanent staff in care homes using bank/agency staff. Compliance to weekly testing among permanent staff is 80%. Results are for 1,000 simulations in each scenario. Boxplot: middle–median; lower hinge– 25% quantile; upper hinge– 75% quantile; lower whisker = smallest observation greater than or equal to lower hinge—1.5 * IQR; upper whisker = largest observation less than or equal to upper hinge + 1.5 * IQR. (DOCX) [file pcbi.1009780.s003.docx]

# S3. Appendix. Additional modelling results

**Table A. Risk of infection in residents and staff in various usage levels of bank/agency staff**

| **Experiment scenario** | **Average usage level of bank/agency staff** | **RR of infection for residents in care homes using bank/agency staff to care homes not using bank/agency staff** | | **RR of infection in bank/agency staff to permanent staff** | | **RR of outbreaks in care homes using bank/agency staff to care homes not using bank/agency staff** | |
| --- | --- | --- | --- | --- | --- | --- | --- |
|  |  | **Compliance to weekly PCR testing among bank/agency staff** | | | | | |
|  |  | **0%** | **80%** | **0%** | **80%** | **0%** | **80%** |
| Different networks | | | | | |  |  |
| A (Heterogeneous size & staff-to-resident ratio) | 5% | 1.68 (1.64 – 1.73) | 1.14 (1.12 – 1.17) | 1.34 (1.29 – 1.38) | 1.28 (1.24 – 1.32) | 2.43 (2.30 – 2.56) | 1.64 (1.54 – 1.73) |
|  | 10% | 2.65 (2.57 – 2.72) | 1.28 (1.25 -1.31) | 1.55 (1.52 – 1.58) | 1.35 (1.32 – 1.38) | 3.76 (3.58 – 3.96) | 1.83 (1.73 – 1.94) |
|  | 15% | 3.73 (3.63 – 3.84) | 1.43 (1.39 – 1.47) | 1.72 (1.69 – 1.75) | 1.42 (1.39 – 1.45) | 4.71 (4.48 – 4.95) | 2.19 (2.08 – 2.32) |
|  | 20% | 5.17 (5.03 – 5.30) | 1.64 (1.60 – 1.68) | 1.98 (1.95 – 2.01) | 1.48 (1.46 – 1.51) | 5.64 (5.37 – 5.92) | 2.48 (2.35 – 2.61) |
| B (Homogeneous size & staff-to-resident ratio) | 5% | 1.65 (1.61 – 1.69) | 1.14 (1.12 – 1.16) | 1.33 (1.29 – 1.37) | 1.29 (1.25 – 1.33) | 2.62 (2.48 – 2.76) | 1.25 (1.17 – 1.33) |
|  | 10% | 2.54 (2.45 – 2.61) | 1.28 (1.25 – 1.31) | 1.52 (1.49 – 1.55) | 1.33 (1.30 – 1.36) | 4.36 (4.14 – 4.59) | 1.82 (1.72 -1.93) |
|  | 15% | 3.72 (3.62 – 3.82) | 1.49 (1.45 – 1.53) | 1.72 (1.69 – 1.75) | 1.45 (1.42 – 1.48) | 5.84 (5.56 – 6.13) | 2.38 (2.25 – 2.52) |
|  | 20% | 5.07 (4.94 – 5.21) | 1.66 (1.57 – 1.69) | 1.98 (1.95 – 2.01) | 1.48 (1.45 – 1.50) | 6.87 (6.55 – 7.21) | 2.83 (2.68 – 2.98) |
| Heterogeneous intra-facility transmission risk drawn from a distribution (Network A) | | | | | |  |  |
| Beta (5, 266) | 5% | 1.75 (1.66 – 1.85) | 1.17 (1.12 – 1.23) | 1.43 (1.39 – 1.48) | 1.32 (1.29 – 1.36) | 2.03 (1.92 – 2.13) | 1.34 (1.27 – 1.42) |
|  | 10% | 2.64 (2.51 – 2.78) | 1.28 (1.22 – 1.35) | 1.62 (1.58 – 1.65) | 1.38 (1.35 – 1.41) | 3.00 (2.85 – 3.14) | 1.50 (1.42 – 1.59) |
|  | 15% | 3.80 (3.62 – 4.00) | 1.56 (1.48 -1.64) | 1.84 (1.80 – 1.87) | 1.56 (1.52 – 1.59) | 3.80 (3.63 – 3.98) | 1.95 (1.85 – 2.06) |
|  | 20% | 5.02 (4.79 – 5.27) | 1.69 (1.60 -1.78) | 2.06 (2.02 – 2.10) | 1.59 (1.56 – 1.62) | 4.51 (4.31 – 4.72) | 2.11 (2.00 – 2.22) |
| Beta (2, 117) | 5% | 1.65 (1.53 – 1.78) | 1.15 (1.06 – 1.24) | 1.64 (1.58 – 1.69) | 1.53 (1.49 – 1.57) | 1.90 (1.80 – 2.00) | 1.35 (1.27 – 1.43) |
|  | 10% | 2.34 (2.17 – 2.52) | 1.21 (1.13 – 1.29) | 1.76 (1.71 – 1.80) | 1.60 (1.56 – 1.64) | 2.61 (2.48 – 2.74) | 1.48 (1.40 – 1.56) |
|  | 15% | 3.12 (2.90 – 3.35) | 1.37 (1.27 – 1.49) | 1.91 (1.87 – 1.95) | 1.61 (1.56 – 1.66) | 3.21 (3.06 – 3.36) | 1.68 (1.59 – 1.77) |
|  | 20% | 3.99 (3.73 – 4.28) | 1.71 (1.57 – 1.85) | 2.12 (2.08 – 2.16) | 1.75 (1.71 – 1.79) | 3.85 (3.67 – 4.03) | 2.02 (1.92 – 2.13) |


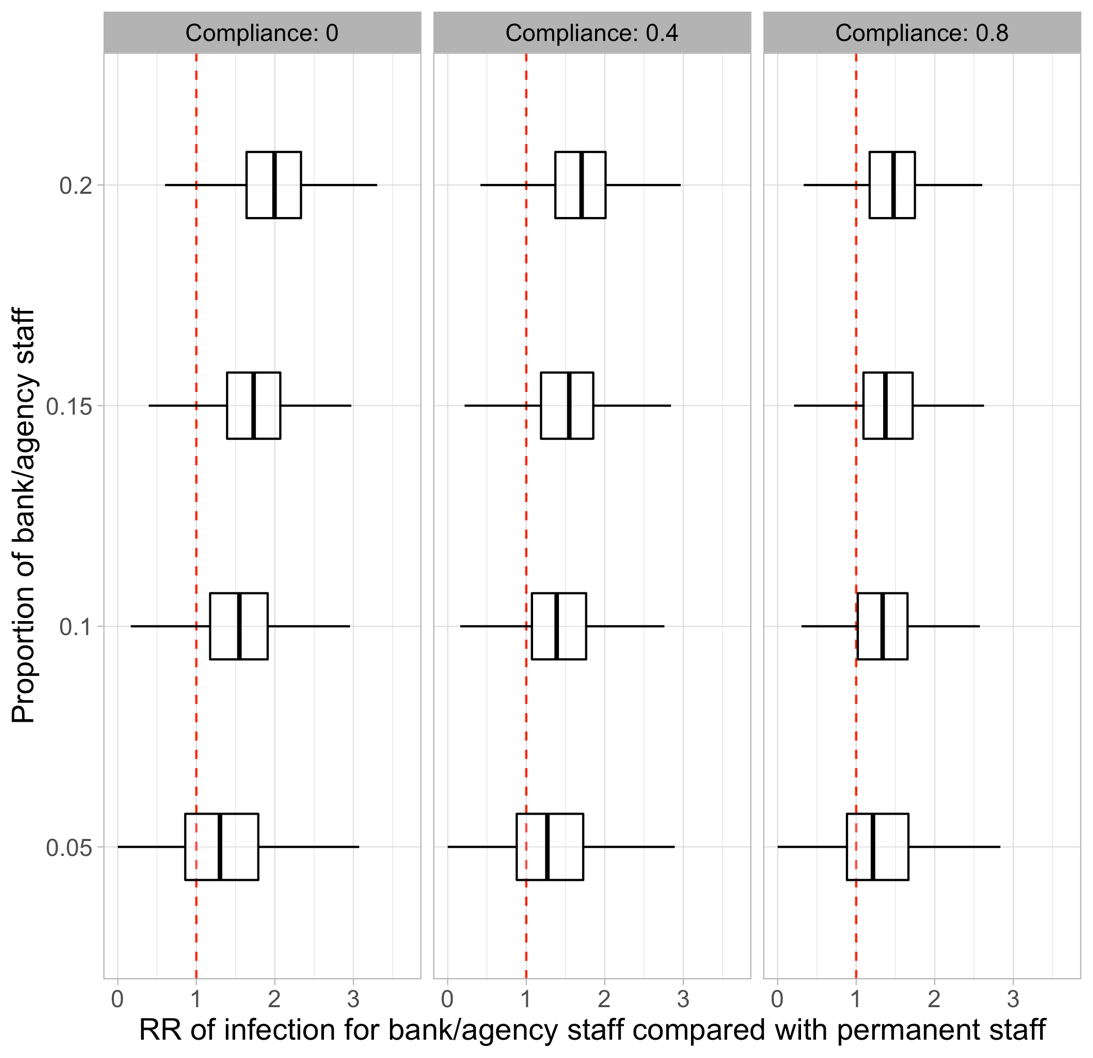


**Fig A. Relative risk [RR] of infection for bank/agency staff with different compliance rates to weekly PCR testing to permanent staff in care homes using bank/agency staff**

Compliance to weekly testing among permanent staff is 80%. Results are for 1,000 simulations in each scenario. Boxplot: middle – median; lower hinge – 25% quantile; upper hinge – 75% quantile; lower whisker = smallest observation greater than or equal to lower hinge - 1.5 * IQR; upper whisker = largest observation less than or equal to upper hinge + 1.5 * IQR
