# Supplementary material for: Hybrid simulation modelling of networks of heterogeneous care homes and the inter-facility spread of Covid-19 by sharing staff
Source: PLoS Comput Biol. 2022 Jan 12;18(1):e1009780. doi: 10.1371/journal.pcbi.1009780 (PMC8789158; doi:10.1371/journal.pcbi.1009780)
Supplement: S4 Appendix — Table A. Output from Partial Rank Correlation Coefficient analyses. Fig A. Impact of staff-to-resident ratio and resident population size on risk of outbreak. The plot describes the risk of outbreak occurrence within 90 days in individual care homes with A: the same population size of 65 residents but different staff-to-resident ratios (network C). B: different resident population size (network D). The average intra-facility transmission risk in care homes is homogeneous. The average usage level of bank/agency staff is 10% of total staff. No intervention on bank/agency staff is implemented. The risk of outbreak occurrence (point) is the probability of simulations where outbreaks occur in 1,000 simulation for each scenario. Line range denotes the 95% CI of this outcome. (DOCX) [file pcbi.1009780.s004.docx]

# S4. Appendix. Results of sensitivity and uncertainty analyses

**Table A. Output from Partial Rank Correlation Coefficient analyses**

| Parameter | Cumulative number of infected residents | | Relative risk of infection in bank/agency staff to permanent staff | |
| --- | --- | --- | --- | --- |
|  | **PRCC** | **p-value** | **PRCC** | **p-value** |
| Community incidence | 0.68 | 3.81E-45 | 0.17 | 6.06E-316 |
| IFR for residents | -0.01 | 2.77E-02 | 0.01 | 1.75E-01 |
| IFR for staff | 0.00 | 7.18E-01 | 0.00 | 9.04E-01 |
| Average resident- resident contact rate | 0.12 | 1.21E-165 | 0.05 | 1.43E-26 |
| Average staff-staff contact rate | 0.07 | 9.16E-51 | 0.05 | 6.76E-31 |
| Average staff-resident contact rate | 0.69 | 5.25E-83 | 0.47 | 1.12E-58 |
| Staff turnover | 0.00 | 6.66E-01 | 0.00 | 3.65E-01 |
| Resident leaving rate | 0.00 | 4.12E-01 | 0.00 | 3.21E-01 |
| Probability of symptomatic in infected residents | -0.14 | 3.32E-231 | -0.12 | 3.60E-149 |
| Probability of symptomatic in infected staff | -0.30 | 1.55E-10 | -0.12 | 1.13E-149 |
| Per-contact transmission probability | 0.91 | 1.79E-241 | 0.77 | 1.50E-47 |
| Pre-symptomatic time | 0.58 | 7.96E-102 | 0.43 | 2.57E-104 |
| Infectious time | 0.06 | 1.50E-47 | 0.05 | 4.62E-27 |
| Social distancing compliance rate | -0.10 | 2.57E-104 | -0.10 | 1.75E-114 |
| PCR sensitivity | 0.00 | 8.68E-01 | 0.00 | 7.43E-01 |
| Test turnaround time | 0.07 | 3.45E-52 | 0.03 | 1.03E-11 |

A negative value indicates a negative correlation – increasing the parameter decreases the outcome. A positive value indicates a positive correlation – increasing the parameter increases the outcome. In PRCC analysis in general, the parameters with large PRCC values (>0.5 or <– 0.5) and corresponding small p-values (<0.05) are deemed the most influential in the model.

The effect of the heterogeneity of resident population size was more significant than staff-to-resident ratio; whilst the latter had no impact on the risk of outbreak across care homes. Decreasing staff-to-resident ratio increased the number of contacts with residents per staff member as the average number of contacts with staff per resident remained the same. This is based on our model assumption that the number of contacts with staff per resident per day was maintained regardless of the staffing level as the overall care home workload does not change. Increasing the number of per-staff-member contacts with residents increased the force of infection in staff which in turn increased the force of infection in residents. However, decreasing staff-to-resident ratio reduced the risk of Covid-19 ingress as fewer staff members enter the care home each day. Overall, the staff-to-resident ratio had no impact on the risk of outbreak in care homes (Fig S4-1A). Our model assumption that staff-to-resident ratio did not affect the per-contact transmission risk or staff’s compliance to other infection control measures may underestimate the impact of this parameter on the risk of outbreak in care homes. Larger care homes had an increased risk of Covid-19 ingress compared with smaller care homes (Figure S4-1B).


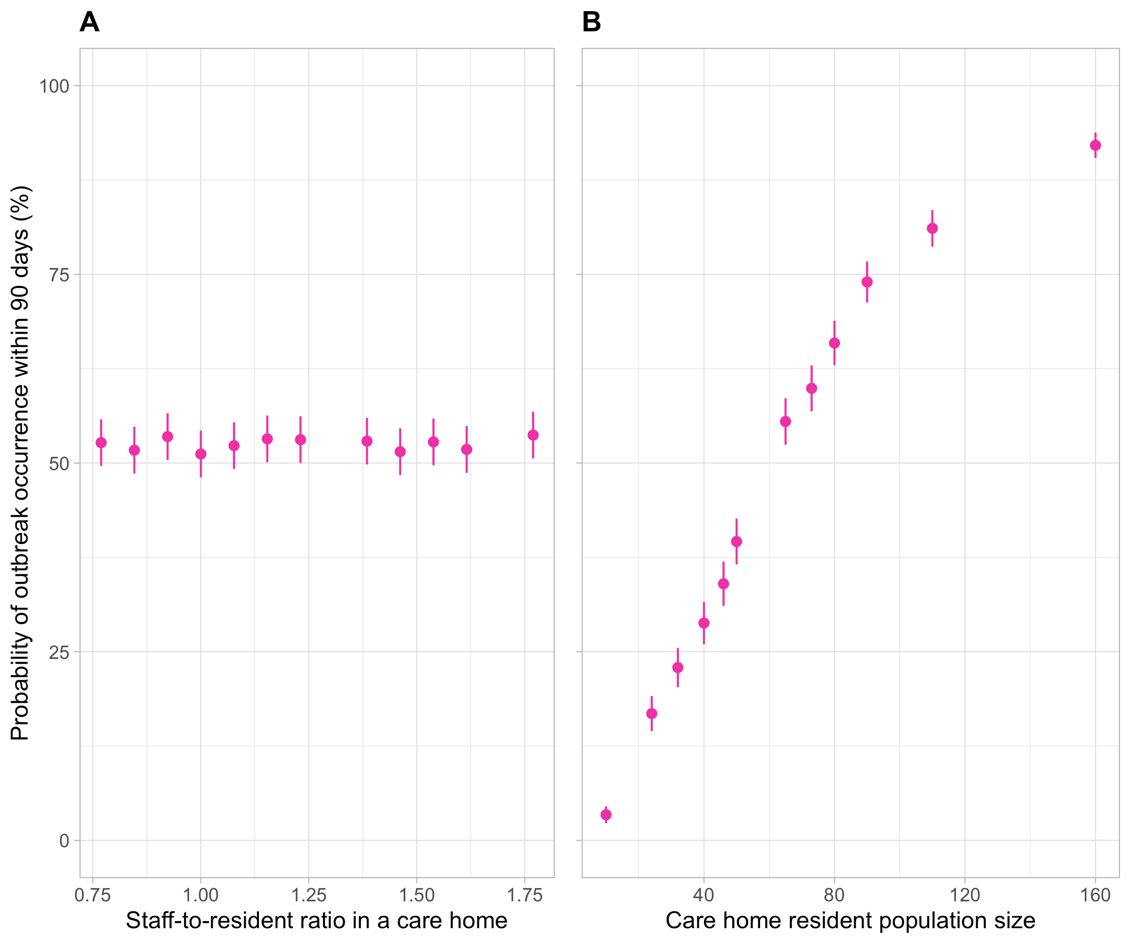


**Fig A. Impact of staff-to-resident ratio and resident population size on risk of outbreak**

The plot describes the risk of outbreak occurrence within 90 days in individual care homes with

A: the same population size of 65 residents but different staff-to-resident ratios (network C).

B: different resident population size (network D).

The average intra-facility transmission risk in care homes is homogeneous. The average usage level of bank/agency staff is 10% of total staff. No intervention on bank/agency staff is implemented. The risk of outbreak occurrence (point) is the probability of simulations where outbreaks occur in 1,000 simulation for each scenario. Line range denotes the 95% CI of this outcome.
