# Supplementary material for: Hybrid simulation modelling of networks of heterogeneous care homes and the inter-facility spread of Covid-19 by sharing staff
Source: PLoS Comput Biol. 2022 Jan 12;18(1):e1009780. doi: 10.1371/journal.pcbi.1009780 (PMC8789158; doi:10.1371/journal.pcbi.1009780)
Supplement: Attachment — Submitted filename: Review Report.pdf [file pcbi.1009780.s005.pdf]

Comments on the manuscript PCOMPBIOL-D-21-01703 "Hybrid simulation modelling networks of heterogeneous care homes and the inter-facility spread of Covid-19 by sharing staff" by Nguyen, Megiddo and Howick.

In this paper, the authors developed a hybrid simulation model that combines system dynamics and agent-based approaches to examine the spread of COVID-19 across multiple care homes via sharing bank/agency staff and evaluate the effectiveness of interventions targeting them. In particular, they calculated the relative infection risk for residents in care homes with and without using bank/agency staff and compared the risk of infection between bank/agency staff and permanent staff. This research topic is relevant and the manuscript can be considered for publication in the journal once the following major comments are addressed.

1. In the Introduction, a comprehensive literature review on COVID-19 modelling, combining system dynamics and agent-based modelling and disease transmission in care home settings is required. For example, there exist quite a few works on combining system dynamics and agent-based modeling such as https://doi.org/10.18564/jasss.4421, https://doi.org/10.1109/WSC.2007.4419767, https://doi.org/10.3390/modelling2020012 and https://doi.org/10.3389/fenvs.2015.00066. The studies on hospital-acquired infections for SARS-CoV-2 or other microorganisms among patients, health-care workers, and caretakers is related.

2. The current study used data from care homes in Lanarkshire as a case study for parametrization. Why did the authors select this location? A little more information about the county is necessary. Do the main conclusions throughout this paper rely on the data of Lanarkshire? In other words, are the main results valid for other countries like the United Sates or some underdeveloped countries? If not, specify the scope of application in the abstract and clarify the possible limitations in the discussion. All residents and staff were assumed to be susceptible at the beginning of simulations, but what happens if mass immunization programs towards residents and staff are implemented?

3. The whole manuscript plus the appendices are quite long. To increase the readability, the manuscript needs to be better organized and presented to give readers a clearer picture and make it easy to follow.

4. It is unclear whether the first paragraph of the introduction is relevant to COVID-19 until one checks the cited papers. Change "Covid-19" to "COVID-19" and give the full name where it first appears. Line 65, "Covid-19. [3]" to "COVID-19 [3]". Lines 206-218, give a little more explanation of the parameter a, what is the definition of $Q_{S,i}$? the number of self-isolated permanent staff members in care home i? Line 227, what is "$WorkRecord[i]$"? Page 14, give appropriate references for the statistical analysis tests or methods if some of them are not well-known to general researchers.
