# Supplementary material for: Hybrid simulation modelling of networks of heterogeneous care homes and the inter-facility spread of Covid-19 by sharing staff
Source: PLoS Comput Biol. 2022 Jan 12;18(1):e1009780. doi: 10.1371/journal.pcbi.1009780 (PMC8789158; doi:10.1371/journal.pcbi.1009780)
Supplement: Attachment — Submitted filename: Review_Reports___PCOMPBIOL_D_21_01703.pdf [file pcbi.1009780.s006.pdf]

Report: PCOMPBIOL-D-21-01703

The allocation of care home for people in need during city lockdown is an important issue for controlling COVID-19. On one hand, temporary staffs are essential when worker shortage is a problem. On the other hand, the mobility of these staffs increases the risk of disease spreading within and between care homes. In the manuscript titled "Hybrid simulation modelling networks of heterogeneous care homes and the inter-facility spread of Covid-19 by sharing staff", the authors utilized a three-layer stochastic model—ABM for the temporary staff allocation and illness progression of temporary staffs, ABM for the group of care homes, and Stochastic Systematic models (SD) for residents and staffs within a certain care home–to evaluate the impacts of different strategic policies. In my opinion, the novelty is that the authors proposed a "multiscale"-like modelling approach which combine two types of mechanistic models and used this approach to simulate the transmission dynamics among care homes in Lanarkshire and assess the effect of different policies. At the end, I recommend for publication after a *major revision*.

Major issues:

1. Why the progression of illness once infected differs between bank/agency staffs and residents/permanent staffs? The asymptomatic and presymptomatic-symptomatic residents and permanent staffs are aggregated into "infectious" in the SD module, while bank/agency staffs experience these stages in illness progression. Why not making the progression consistent?

2. In the Table S1-5, it seems that the transmissbility of all infectious individuals are the same, however, the transmissibility of asymptomatic patients is much weaker [1].

3. Due to my ignorance, can the author explain how to model the $DELAY()$ function in the SD model? To my understanding, when modelling the dynamical system using small time step, it's hard to ensure the exact time delay in the model. I am eager to learn this technique.

4. In Outcomes: why is the 95% CI symmetric? Can the authors justify the construction of 95% CI with details?

5. I noticed that for most of the parameters used in the model (Table S1-4), triangular distributions are used for sensitivity analyses. Can the authors explain why triangular distribution instead of others (*i.e.,* gamma, beta, *etc.*).

Minor issues:

1. In Fig 1, I think it should be "Intra facility Module - SD".

2. In Line 363 on Page 14, that should be "Table S1-4", not "Table 2".

3. In Line 386 and 387 on Page 18, please provide references for the parameters used in Beta() distributions.

References

[1] Byambasuren O, Cardona M, Bell K, Clark J, McLaws ML, Glasziou P. Estimating the extent of asymptomatic COVID-19 and its potential for community transmission: Systematic review and meta-analysis. J Assoc Med Microbiol Infect Dis Can. 2020;5:223-34.
