# Supplementary material for: Hybrid simulation modelling of networks of heterogeneous care homes and the inter-facility spread of Covid-19 by sharing staff
Source: PLoS Comput Biol. 2022 Jan 12;18(1):e1009780. doi: 10.1371/journal.pcbi.1009780 (PMC8789158; doi:10.1371/journal.pcbi.1009780)
Supplement: Attachment — Submitted filename: Response to Reviewers_PlosCB.docx [file pcbi.1009780.s007.docx]

November 26^th^, 2021

Dr. Virginia Pitzer (Deputy Editor-in-Chief)

Dr. Claudio José Struchiner (Associate Editor)

PLOS Computational Biology

Cambridge, UK

Dear Dr. Pitzer and Dr. Struchiner,

We are pleased to submit a revised version of our manuscript “Hybrid simulation modelling networks of heterogeneous care homes and the inter-facility spread of Covid-19 by sharing staff” for consideration at PLOS Computational Biology.

We appreciate the helpful comments from reviewers. We have addressed the specific concerns in the following pages.

We look forward to hearing from you.

Your sincerely,

Le Khanh Ngan Nguyen

B.Pharm, MPH, Doctoral Researcher

Department of Management Science

University of Strathclyde

E:[Nguyen-le-khanh-ngan@strath.ac.uk](mailto:Nguyen-le-khanh-ngan@strath.ac.uk)

**Reviewer #1:**

**Comment 1.1:** This paper develops a model of the spread of covid infections in care homes and it explores a number of relevant scenarios related to staffing, with particular interest to the use of bank staff. This is a topical issue with the potential to inform decisions in staffing of care homes and it is worthy of publication. The paper is well designed and written and the models well implemented. Some suggestions for further improvement are listed below:

**Response 1.1: Thank you.**

**Comment 1.2:** The paper discusses the use of hybrid simulation in the introduction. At this point the justification of using a hybrid model does not work very well. It refers to the use of hybrid modelling, but it is not clear for the reader why hybrid simulation is suitable. It would be better if the authors explain in the introduction the problem and the key aspect that the model needs to represent. The justification of the use of hybrid modelling could be discussed in the materials and methods section.

**Response 1.2: We discussed the reasons for using hybrid simulation modelling in general in the Introduction (“Each simulation … methods together”, lines 107 – 113). We agree that adding more specific reasoning for this paper benefits the reader, and we add a brief explanation to justify the use of a hybrid model for our study in the Introduction section (“We find … care home.”, lines 142 – 147) and the Material and Methods section (“… as it is … modelling method.”, lines 175 – 177). We also clarify that we have explained the choice of an appropriate simulation modelling method for each module in detail in the sub-sections Network module, Temporary staff module, and Intra-facility module (“We also explain … for each module.”, lines 181 – 182).**

**Comment 1.3:** It seems like the problem is the lack of staff in care homes, which is only indirectly referred to in the paper. It would be useful to make this clearer from the beginning and in the abstract also.

**Response 1.3: We have added the explanation about staff shortages and why bank/agency staff are needed in the Abstract (“Care homes … staff shortages.”, lines 24 – 25). We also explain this in the Author summary (“Our model findings … shortages of staff.”, lines 47 – 49) and the Introduction (“Furthermore, care homes … amid the pandemic”, lines 75 – 78).**

**Comment 1.4:** What is the aim of the model? It would be useful to include in the methods section the aim and questions that the model aims to answer.

**Response 1.4: We explain the aims of the model in the Introduction section (“In this study, we develop … these impacts”, lines 136 – 138, and, “It also …network compositions”, lines 149 – 150). We agree that we can make this clearer and have slightly amended this sentence to highlight the aims of the model. We also include the modelling aims in the first section “Purpose and Patterns” of the Overview, Design concepts, and Details (ODD) protocol (Appendix S1).**

**Comment 1.5:** Results section - Impact of different usage levels of bank/agency staff. It would be useful to indicate whether there is a statistically significant difference in the RR of infection or outbreak between the different scenarios tested.

**Response 1.5: We have added this clarification (“There was … scenarios tested.”, lines 444 – 445). We have also slightly amended the topic sentence to emphasize the statistically significant impact of the usage level of bank/agency staff on these outcomes (The usage level … across care homes”, lines 442 – 443).**

**Comment 1.6:** There are two sections on validation - Confidence building (Verification and validation) and Validation results. Consider merging.

**Response 1.6: The first sub-section “Confidence building (verification and validation)” under the Methods section describes different approaches that we have adopted to build confidence in the model. As the methods of building confidence in hybrid simulation models are not commonly reported in the literature (Brailsford et al. 2019), we explicitly explain these approaches to highlight the contribution of the paper. The second sub-section “Validation results” summarizes the results for validation, and therefore, belongs to the Results section. We have adjusted the heading of the first sub-section to “Confidence building approach” to reinforce this difference.**

**Brailsford, S. C., Eldabi, T., Kunc, M., Mustafee, N. & Osorio, A. F. 2019. Hybrid simulation modelling in operational research: A state-of-the-art review. *European*** ***Journal of Operational Research,* 278, 721-737.**

**Comment 1.7:** There are some typos in the manuscript that need attention. For example line 80 – understaffed instead of understaffing. Please check the manuscript carefully.

**Response 1.7: We have revised the manuscript and corrected the typos**

**Comment 1.8:** Overall, i think this is very interesting work and worthy of publications. I look forward to seeing the revised manuscript.

**Response 1.8: Thank you for your helpful comments**

**Reviewer #2:**

**Comment 2.1:** This is a rather comprehensive study with complex modelling consideration on the disease transmission in a heterogeneously mixed system.

**Response 2.1: Thank you.**

**Comment 2.2:** I have the following concerns. The major one is that this manuscript seems to lack a real-world dataset to support the modelling outcomes.

**Response 2.2: The model parameters and structure are based on real-world data. We adopt multiple approaches to collect data: literature review, discussion and interviews with several care home stakeholders, and data provided by care homes and the Health and Social Care Partnerships Lanarkshire. We explicitly reference the source of data for the model parameters in Table S1-4 and throughout the manuscript.**

**Although we do not compare the modelling results to a real-world time series, we build confidence in the robustness of the modelling outcomes using several verification and validation approaches (“Confidence building approach” under “Methods”, lines 360 – 439). Specifically, in our black-box validation, we adopt the pattern-oriented modelling approach (Grimm et al, 2005) to assess the model’s ability to reproduce the patterns observed in real-world (“In black-box … bank/agency staff.”, lines 388 – 401).**

**Additionally, obtaining real-world time series data to compare the output against is difficult. Firstly, we rely on data of infections among residents and staff provided by care homes. Data collection is likely to be inconsistent across care homes and over time within a care home. The number of infections are likely to be underreported due to asymptomatic infections, staff’s imperfect compliance to weekly testing, and weekly testing of residents not being implemented. Additionally, data on the number of infections among bank/agency staff are not available. Also, during the pandemic, changes in the usage level of bank/agency staff have been highly uncertain and vary across care homes. Some care homes report that they have adopted measures to reduce/halt the use of bank/agency staff and some bank/agency staff have also refused to work in care homes which are experiencing an outbreak.**

**Furthermore, even if this data is readily available, the paper does not aim to reproduce a specific realisation of the historical epidemic curve, but to understand how care home and network characteristics affect that curve (and morbidity and mortality) and the relative effectiveness of interventions. We also explore the consistency of findings on the relative effectiveness of different interventions targeting bank/agency staff in the uncertainty analysis. We discuss this in the Discussion (“Several key … parameters and structures.”, lines 730 – 741).**

**Grimm V, Revilla E, Berger U, Jeltsch F, Mooij WM, Railsback SF, et al. (2005). Pattern-Oriented Modeling of Agent-Based Complex Systems: Lessons from Ecology. Science;310(5750):987.**

**Comment 2.3:** in table S1-5, the F*xi term in the transmission path seems strange to me. what is the meaning of (1 - F*xi)? And what is the intuition behind this term? Besides, it seems the physical unit does not hold consistently. The unit of all ‘flow’ should be ‘# of individuals’, right?

**Response 2.3: In the module for the intra-facility transmission dynamics, we develop a stochastic system dynamics model by introducing the heterogeneous parameter noise (**$\boldsymbol{F}_{\boldsymbol{i}}\boldsymbol{\xi}_{\boldsymbol{i}}$**) directly into the standard differential equations. We do not use** $\boldsymbol{(1-F}\boldsymbol{\xi)}$**, however, when introducing this noise to the original equation denoted** $\frac{\boldsymbol{dI}}{\boldsymbol{dt}}\boldsymbol{= \beta}\frac{\boldsymbol{SI}}{\boldsymbol{N}}$**, the equation becomes** $\frac{\boldsymbol{dI}}{\boldsymbol{dt}}\boldsymbol{= \beta}\frac{\boldsymbol{SI}}{\boldsymbol{N}}\boldsymbol{+\beta F}\frac{\boldsymbol{SI}}{\boldsymbol{N}}\boldsymbol{\xi}$ **with** $\boldsymbol{F=}\frac{\boldsymbol{1}}{\sqrt{\boldsymbol{I}}}$ **(Keeling and Rohani, 2008, p194 – 200). This formulation is equal to** $\frac{\boldsymbol{dI}}{\boldsymbol{dt}}\boldsymbol{=\beta(1+F}\boldsymbol{\xi)}\frac{\boldsymbol{SI}}{\boldsymbol{N}}$**.**

**The noise (dimensionless) represents parameter fluctuations caused by individual variation, both in terms of the pattern of contacts and the immunological response to infection (Keeling and Rohani, 2008). We have added this explanation into Table S1-5 where the term** $\boldsymbol{F}_{\boldsymbol{i}}\boldsymbol{\xi}_{\boldsymbol{i}}$ **first appears (“**$\boldsymbol{F}_{\boldsymbol{i}}\boldsymbol{\xi}_{\boldsymbol{i}}$**: heterogeneous parameter noise, representing parameter fluctuations caused by individual variation (unitless)”). We also refer to Keeling and Rohani (2008) (Reference #43) which provides details on the methods of modelling stochastic dynamics.**

**Generally, the unit for the flows is persons/time. Specifically, the unit of the flow** $\frac{\boldsymbol{d}\left( \boldsymbol{I}_{\boldsymbol{R}} \right)}{\boldsymbol{dt}}$ **, for example, is infected residents/time. The units for both sides of each equation are consistent.**

**Keeling MJ, Rohani P. *Modeling Infectious Diseases in Humans and Animals.* Princeton University Press; 2008.**

**Comment 2.4:** should it be normal (0,1)? Normal (1,0) means just constant 1.

**Response 2.4: Yes, it is Normal (mean = 0, variance = 1). Thank you for highlighting this error, we have corrected it.**

**Comment 2.5:** is there any fitting conducted in this study? If yes, how is the fitting conducted? So far, I did not see the fitting results.

**Response 2.5: We do not conduct fitting in this study. Our model does not aim to make inference about relationships between variables in given data sets nor make predictions of future outcomes based on models estimated using historical data. Rather, the modelling objective is to explore and understand how care home and network characteristics affect the epidemic curve and to assess the relative impact of interventions. Please see Response 2.2, where we explain this in detail. We also do not have a complete historical data set for fitting due to the difficulties of obtaining such a data set as explained in Response 2.2. As mentioned above, we use several verification and validation approaches to build confidence in the model. Specifically, we adopt a pattern-oriented modelling approach in black-box validation to assess the model’s ability to reproduce the patterns observed in and across care homes in the UK (“In black-box validation … via bank/agency staff.”, lines 388 – 401).**

**Comment 2.6:** please discuss the limitations of this study.

**Response 2.6: We discuss the limitations of this study in the second last paragraph of the “Discussion” section (“Our modelling study has a number of limitations. … outbreaks may decrease over time.”, lines 705 – 729)**

**Reviewer #3:**

**Comment 3.1:** In this paper, the authors developed a hybrid simulation model that combines system dynamics and agent-based approaches to examine the spread of COVID-19 across multiple care homes via sharing bank/agency staff and evaluate the effectiveness of interventions targeting them. In particular, they calculated the relative infection risk for residents in care homes with and without using bank/agency staff and compared the risk of infection between bank/agency staff and permanent staff. This research topic is relevant and the manuscript can be considered for publication in the journal once the following major comments are addressed.

**Response 3.1:** **Thank you.**

**Comment 3.2:** In the Introduction, a comprehensive literature review on COVID-19 modelling, combining system dynamics and agent-based modelling and disease transmission in care home settings is required. For example, there exist quite a few works on combining system dynamics and agent-based modeling such as, <https://doi.org/10.1109/>WSC.2007.4419767, https://doi.org/10.3390/modelling2020012 and <https://doi.org/10.3389> /fenvs.2015.00066. The studies on hospital-acquired infections for SARS-CoV-2 or other microorganisms among patients, health-care workers, and caretakers is related.

**Response 3.2: We add a literature review on hybrid SD-ABM models studying healthcare-associated infections and the spread of COVID-19 (“Hybrid simulation … computational costs”, lines 120 – 135). Most of the studies on healthcare-associated infections focus on acute care settings while neglecting care homes.**

**Comment 3.3:** The current study used data from care homes in Lanarkshire as a case study for parametrization. Why did the authors select this location? A little more information about the county is necessary. Do the main conclusions throughout this paper rely on the data of Lanarkshire? In other words, are the main results valid for other countries like the United Sates or some underdeveloped countries? If not, specify the scope of application in the abstract and clarify the possible limitations in the discussion. All residents and staff were assumed to be susceptible at the beginning of simulations, but what happens if mass immunization programs towards residents and staff are implemented?

**Response 3.3: We have added background information about care homes in Lanarkshire in the sub-section “Base-case care home data” under “Materials and methods” (lines 159 – 173). We discuss the robustness of the model findings to uncertainty in model parameters and structure (“Several key parameters … parameters and structure.”, lines 730 – 741). Our base case model reflects the characteristics of care homes in the UK but could be tailored to a specific network of care homes in other countries to evaluate impacts of policies targeting staff working across multiple facilities. This can be done by adjusting the inputs characterising resident population size, staffing level and bank/agency staff usage level of care home agents, and by modifying the work scheduling rules for bank/agency staff agents.**

**The question about what happens if mass immunization programs towards residents and staff are implemented is very interesting and worth exploring, in future work.**

**Comment 3.4:** The whole manuscript plus the appendices are quite long. To increase the readability, the manuscript needs to be better organized and presented to give readers a clearer picture and make it easy to follow.

**Response 3.4: Our study investigates a complex problem using a multiscale hybrid simulation modelling approach and, therefore, it requires explicit explanations. The length of the manuscript follows the submission guideline of PLOS Computational Biology. We divide the paper into sections and sub-sections with clear headings that follow the submission guidelines of the journal to enhance readability. The purpose of the ODD protocol (Appendix S1) is for reproducing the model and not all audiences need to necessarily read it to understand the study.**

**Comment 3.5:** It is unclear whether the first paragraph of the introduction is relevant to COVID-19 until one checks the cited papers. Change “Covid-19" to “COVID-19" and give the full name where it first appears.

**Response 3.5: We have made these changes as you suggest. We also add the full name of the coronavirus disease (COVID-19) in the first paragraph of the Introduction (line 60).**

**Comment 3.6:** Line 65, “Covid-19. [3]" to “COVID-19 [3]".

**Response 3.6: We have corrected the above as suggested.**

**Comment 3.7:** Lines 206-218, give a little more explanation of the parameter a, what is the definition of QS;i? the number of self-isolated permanent staff members in care home i? Line 227, what is “WorkRecord[i]"?

**Response 3.7:** $\boldsymbol{Q}_{\boldsymbol{S,i}}$ **denotes the number of permanent staff members in care home *i* who have to self-isolate due to COVID-19. We have amended this for clarification (lines 259 – 260).**

***WorkRecord[i]* is a bank/agency staff specific state variable and denotes the number of times that the staff member works in care home *i.* We have added the definition of this variable into the manuscript (lines 269 – 271). We also include this in Table S1-1 of Appendix S1 that defines all of the state variables of care home agents and bank/agency staff agents.**

**Comment 3.8:** Page 14, give appropriate references for the statistical analysis tests or methods if some of them are not well-known to general researchers.

**Response 3.8: We have added the reference for the adopted statistical analysis approaches (Reference [45], line 357).**

**Reviewer #4:**

**Comment 4.1:** The allocation of care home for people in need during city lockdown is an important issue for controlling COVID-19. On one hand, temporary staffs are essential when worker shortage is

a problem. On the other hand, the mobility of these staffs increases the risk of disease spreading

within and between care homes. In the manuscript titled “Hybrid simulation modelling networks

of heterogeneous care homes and the inter-facility spread of Covid-19 by sharing staff”, the authors

utilized a three-layer stochastic model—ABM for the temporary staff allocation and illness progression of temporary staffs, ABM for the group of care homes, and Stochastic Systematic models (SD) for residents and staffs within a certain care home–to evaluate the impacts of different strategic policies. In my opinion, the novelty is that the authors proposed a “multiscale”-like modelling approach which combine two types of mechanistic models and used this approach to simulate the transmission dynamics among care homes in Lanarkshire and assess the effect of different policies. At the end, I recommend for publication after a major revision.

**Response 4.1: Thank you.**

**Comment 4.2:** Major issues:

1. Why the progression of illness once infected differs between bank/agency staffs and residents/

permanent staffs? The asymptomatic and presymptomatic-symptomatic residents and

permanent staffs are aggregated into “infectious” in the SD module, while bank/agency staffs

experience these stages in illness progression. Why not making the progression consistent?

**Response 4.2: For residents and permanent staff, we implicitly account for the asymptomatic, pre-symptomatic, and symptomatic states in the flows (**$\boldsymbol{Flo}\boldsymbol{w}_{\boldsymbol{I}_{\boldsymbol{R}}\boldsymbol{Q}_{\boldsymbol{R}}}$ **and** $\boldsymbol{Flo}\boldsymbol{w}_{\boldsymbol{I}_{\boldsymbol{S}}\boldsymbol{Q}_{\boldsymbol{S}}}$ **) to keep the structure of the SD module simple, reduce the number of equations, and keep the equations for the transmission flows simple. We have added this explanation to sub-section 7.4. *Intra-facility* on page 12 in Appendix S1 (“This module implicitly … flows simple.”). The disease progression for bank/agency staff agents is coded consistently using the same sets of parameters as for permanent staff. We have amended Figure S1-2 (by replacing the links between the sub-states “Asymptomatic” and “Symptomatic” and “Recovered” with a link between the state “Infectious” and “Recovered”) and the process *Temporary_Staff_Disease_Progression* (under section 3. Process Overview and Scheduling, p4) in Appendix S1 to clarify this consistency.**

**Comment 4.3:** In the Table S1-5, it seems that the transmissibility of all infectious individuals are the same, however, the transmissibility of asymptomatic patients is much weaker [1].

References

[1] Byambasuren O, Cardona M, Bell K, Clark J, McLaws ML, Glasziou P. Estimating the extent

of asymptomatic COVID-19 and its potential for community transmission: Systematic review

and meta-analysis. J Assoc Med Microbiol Infect Dis Can. 2020;5:223-34.

**Response 4.3: Our model assumes that the transmissibility of all infectious individuals is the same (“It is assumed that pre-/asymptomatic individuals are just as likely to transmit infection as symptomatic individuals …”, p5 in Appendix S1) for the following reasons. Firstly, the difference in transmissibility is not important for the purpose of the model and the interventions that we examine. Thus, we assume the same transmissibility for simplification. Secondly, we assume that care home residents and staff will go into isolation after developing symptoms and can no longer spread the disease. This assumption is based on government guidance and insight from care homes that residents are monitored daily for COVID-19 symptoms during the pandemic. We assume that infected individuals who have mild or atypical symptoms and, therefore, cannot be detected quickly enough, are considered asymptomatic. Therefore, all transmission events are caused by individuals who are asymptomatic or pre-symptomatic. We assume perfect effectiveness of resident isolation in the model (Table S1-5). Care homes report that staff that care for symptomatic residents are not mixed with other staff and residents. They also have to follow contact precautions strictly and this would reduce the transmission probability per contact.**

**Comment 4.4:** Due to my ignorance, can the author explain how to model the DELAY() function in the SD model? To my understanding, when modelling the dynamical system using small time step,

it’s hard to ensure the exact time delay in the model. I am eager to learn this technique.

**Response 4.4: The DELAY() function in the SD module is a discrete or pipeline delay and takes the form: DELAY(input, delayTime, initialValue). Use of this function means that all delays that it creates take exactly the same length of time, which is delayTime. However, until delayTime is reached, the function will return the initialValue. We have added this clarification to where the DELAY() function first appears (Table S1-5, Appendix S1). The delay time in the SD module is the average delay time for deaths and recovery (individuals are in isolation during this period). The time step** $\boldsymbol{\delta}$**t^-^ is 1/2^7^ days (Table S1-2 in Appendix S1) and as the delay time is a multiple of the time step, the exact delay time can be ensured in the model.**

**Comment 4.5:** In Outcomes: why is the 95% CI symmetric? Can the authors justify the construction of 95% CI with details?

**Response 4.5: Not all CIs in the paper are symmetric. It depends on the estimate. Specifically, the CIs for the mean number of infections are symmetric, and are calculated following the Central Limit Theorem stating that the sampling distribution of the sample means will approach a normal distribution as the sample size increases (n=1,000). The CIs for the probability of outbreak occurrence (also symmetric) are constructed using the conventional “Wald” interval that employs the Normal approximation to the Binomial distribution as the number of simulations is large. The CIs for relative risk of outbreak occurrence are not symmetric, and are calculated using the method described in Morris and Gardner (1988). This 95%CI is asymmetric as shown in Figure 3.**

**Morris, J. A., & Gardner, M. J. (1988). Calculating Confidence Intervals For Relative Risks (Odds Ratios) And Standardised Ratios And Rates. *British Medical Journal (Clinical Research Edition)*, *296*(6632), 1313–1316. http://www.jstor.org/stable/29530653**

**Comment 4.6:** I noticed that for most of the parameters used in the model (Table S1-4), triangular distributions are used for sensitivity analyses. Can the authors explain why triangular distribution

instead of others (i.e., gamma, beta, etc.).

**Response 4.6: We use triangular distributions for parameters where the literature and other data sources provide information on what the minimum, maximum, and most likely values are. However, such information is insufficient to determine what the distributions for these parameters are. We include the references for the distributions of parameters used in the model in Table S1-4. In cases we have sufficient information to posit the shape of the distribution, we do so.**

**Comment 4.7:** Minor issues:

1. In Fig 1, I think it should be “Intra facility Module - SD”.

2. In Line 363 on Page 14, that should be “Table S1-4”, not “Table 2”.

3. In Line 386 and 387 on Page 18, please provide references for the parameters used in Beta()

distributions.

**Response 4.7:**

**Thank you for noting these:**

**1. We have amended the above as suggested.**

**2. We have corrected it.**

**3. We have added the citations based on which we choose the Beta distribution shape ([52, 53], line 430). We also explain how we obtain the two Beta distributions (“In the second scenario to reflect … held other parameters constant.”, lines 389 – 400).**
